# Supplementary material for: Patients’ experiences of life after bariatric surgery and follow-up care: a qualitative study
Source: BMJ Open. 2020 Feb 6;10(2):e035013. doi: 10.1136/bmjopen-2019-035013 (PMC7045271; doi:10.1136/bmjopen-2019-035013)
Supplement: Supplementary data [file bmjopen-2019-035013supp001.pdf]

Study Protocol version 2.0 (7/7/14)

Document S1 (Study protocol)

The patient perspective of living with surgery for morbid obesity: Creating a patient 'core' outcome set, and investigating ways to improve follow-up care

Chief Investigator

Karen D Coulman, NIHR Doctoral Research Fellow, School of Social and Community Medicine, Unversity of Bristol

Supervisors

Dr Amanda Owen-Smith, School of Social and Community Medicine, Unversity of Bristol

Professor Jane M Blazeby, School of Social and Community Medicine, Unversity of Bristol

Funding

National Institute of Health Research (NIHR) Doctoral Research Fellowship award

Study Protocol version 2.0 (7/7/14)

Lay Summary

Surgery is increasingly being used as a treatment for obesity. Studies report many different outcomes, often measured in different ways, and little is known about what outcomes are important to patients. This study aims to explore the outcomes that are important to patients and to use this information to develop a short list of the most important outcomes to health professionals and patients (a core outcome set), which can be used to evaluate surgical treatments for obesity. In addition, this study will investigate patients' experiences of obesity surgery to provide recommendations for how post-operative support can be improved in the NHS. This will be achieved by interviewing obesity surgery patients to determine whether published outcomes reflect those outcomes patients themselves consider important, and to obtain views on living with surgery and ways to improve post-operative support. A list of patient-centred outcomes will be created from the literature and the interviews which will be added to a list of 'medical' outcomes of obesity surgery. This long list of outcomes will be reduced into a "core" set of outcomes, using a scientific process to reach agreement, which involves 2 rounds of questionnaires completed by health professionals and patients. The final core outcome set will be agreed in separate consensus meetings with a small group of health professionals and patients. This core outcome set will be useful to researchers in choosing outcomes for research trials that are important to both health professionals and patients. Use of the core outcome set would also improve the overall quality of reporting in trials. This research will also be useful for health professionals to ensure health services are designed based on patient priorities.

Study Protocol version 2.0 (7/7/14)

Background

Obesity surgery

The NHS Information Centre has released figures showing that just over one quarter of the adult population of England was obese in 2010, an increase from 13-16% of the population in 1993[1]. It is predicted that one third of adults in England will be obese by 2015 and more than 700 million adults will be obese worldwide by 2015[1;2]. Obesity is associated with an increased risk of type 2 diabetes, heart disease, cancer, reduced quality of life and premature death[1;3]. As such, obesity is a major public health concern. Various treatments for obesity exist including lifestyle modifications, pharmacotherapy, and more recently, surgical interventions. Obesity surgery operations have rapidly increased in the UK, and worldwide, with the most common operations being the adjustable gastric band and the Roux-en-Y gastric bypass[1;4]. In 2009, a Health Technology Assessment report (including a Cochrane systematic review) concluded that obesity surgery is more clinically effective than other treatments for obesity in terms of weight loss, improvement of co-morbidities such as type 2 diabetes and hypertension, and is also cost effective[5].

The patient perspective of outcomes of obesity surgery

Although clinical outcomes are important to measure, patient-reported outcome (PRO) measures, such as measures of health-related quality of life (HRQL), provide a means for capturing how a patient feels about their health or condition[6]. The Food and Drug Administration (FDA) has published guidance on the development of PRO measures to support claims of treatment benefit[6]. PRO measures should be developed based on rigorous qualitative research with patients to ensure content validity (authenticity to patients)[6;7]. Despite this, the development of PRO measures is variable and not always transparent[7].

The lead researcher has recently undertaken a systematic review of PROs in obesity surgery studies, which identified 68 different validated questionnaires used to assess PROs amongst the 86 identified studies [8]. Due to the heterogeneity of the items and scales within the questionnaires used amongst the various studies, a meta-analysis to determine the effect of obesity surgery on PROs was unable to be undertaken. The lead researcher then looked in further depth at the development of those questionnaires which were classified as obesity-specific (19 questionnaires). Although 14 (74%) reported to have involved patients in questionnaire development, only 3 (16%) specified that obesity surgery patients were involved. Only 6 (32%) specified that qualitative interviews/focus groups were used to obtain patient views and none specified that a qualitative analysis of resulting data was undertaken. Thus, it is not certain whether the majority of PRO questionnaires being used to assess obesity surgery are grounded in patient views.

Although well-developed PRO measures can provide valuable information about the patient's perspective of outcomes of treatment, primary qualitative studies can also yield rich information; however, these types of studies are rarely included in Cochrane reviews of quantitative evidence. The lead researcher is currently undertaking a review of qualitative research studies where the patient perspective of obesity surgery was sought. Although qualitative studies have focused on patient experiences of surgery, none have explicitly investigated patients' views of important outcomes of obesity surgery.

There is clearly more work needed to clarify the outcomes of obesity surgery from the patient perspective.

A 'core' outcome set for obesity surgery

As described above, heterogeneity of PROs in obesity surgery studies is an issue, which limits cross-study comparison and amalgamation of study results, which in turn limits the ability to make recommendations to clinicians and policymakers about the impact of obesity surgery on outcomes

important to patients. This issue is not unique to PROs, but also to clinical outcome measures with a Cochrane review of obesity surgery unable to undertake a meta-analysis due to a lack of consistency in outcomes reported[5].

Recent years have seen an increasing interest in the establishment of 'core outcome measures' to promote consistency in the reporting of clinical trials. Of particular interest is the development of the COMET (Core Outcome Measures in Effectiveness Trials) initiative through the MRC Hubs for Trials Methodology Research whose aim is to bring together researchers interested in the development and application of core outcome sets for specific conditions[9;10](1). Another notable initiative is OMERACT (Outcome Measures in Rheumatology Clinical Trials) in rheumatology[11]. Core outcome sets are not meant to be a restrictive list, but a minimum set of outcomes that should be reported on in every trial of a particular disease/condition. By standardising outcomes to be reported on in trials, results can be compared, contrasted and synthesised (e.g. meta-analysis) to inform health policy. No core outcome set for obesity surgery currently exists.

Recent research demonstrates that patients' perceptions of important outcomes may differ considerably from clinicians' perspectives, and therefore incorporating the patient perspective is crucial to contribute to and validate core outcome sets[12]. Creating a core outcome set for obesity surgery based on both health professional and patient views will help in the ordering of research and health service priorities to include the patient perspective, and ultimately improve long-term outcomes.

The patient perspective of follow-up care after obesity surgery

Follow-up care after obesity surgery varies greatly across centres. There is no consensus as to what optimum follow-up care is, although most clinicians recognise that good follow-up care is integral in achieving good outcomes from obesity surgery[13-15]. Understanding the outcomes of most importance to patients, and their experiences of follow-up care would provide invaluable information to design effective follow-up care programmes tailored to patients needs to maximise the benefits of surgery in the long-term. Qualitative research with obesity surgery patients in the UK context has focused on the experiences of patients, including difficulties they encounter after obesity surgery[16;17] but to our knowledge, no studies have specifically focused on patients' experiences of follow-up care and ways to improve follow-up care in the health services.

There is clearly more work needed to clarify optimum follow-up care from the patient perspective, and its influence on outcomes. As obesity and the number of obesity surgery operations increase, it is imperative that research in this area is of high quality, and takes into account the patient perspective.

Aims

The overall aims are to develop a core outcome set for obesity surgery including both health professional and patient perspectives and to investigate patients' experiences of living with the results of obesity surgery including ways to improve post-operative follow-up care.

Objectives

1. Semi-structured qualitative interviews with obesity surgery patients will be conducted to:
    a. Investigate outcomes of importance to obesity surgery patients that may not have been documented in the literature previously, and to add these to a comprehensive list of PROs of obesity surgery previously compiled from systematic literature reviews.
    b. Investigate patients' experiences of living with obesity surgery and ways to improve post-operative support.

Study Protocol version 2.0 (7/7/14)

2. The list created in objective 1a above will be added to a list of clinical outcomes which will be used within a Delphi survey of health professionals and patients which will ask them to prioritise the outcomes and produce a shorter list. The short list will be considered at separate meetings with health professionals and patients who will agree and finally ratify the content of the core outcome set.

Methods

Semi-structured qualitative interviews

To supplement the literature reviews previously undertaken by the lead researcher, patients' views of important outcomes of obesity surgery and experiences of existing follow up care will be investigated using semi-structured interviews within a qualitative paradigm.

Identification and selection of patients
Patients who are about to undergo and who have undergone a primary operation for obesity will be identified by health professionals from participating obesity surgery services at NHS healthcare Trusts using departmental databases and clinic lists. The main criteria for selection will be: The patient is undergoing obesity surgery within the next three months (pre-surgery group), or has undergone obesity surgery (post-surgery group). Including patients at both the pre- and post-operative stage will allow us to compare views and assess differences. Patients will initially be purposively sampled to obtain maximum variation for gender, age, ethnicity, starting BMI, type of operation, and time since operation, however further sampling will be guided by emerging findings from interviews.

Patient recruitment
Identified patients will be sent a letter from their local NHS obesity surgery team including an invitation letter and patient information sheet from the lead researcher, informing them of the research and inviting them to participate in the interviews. A reply slip and stamped addressed envelope will be included for patients to indicate whether or not they are interested in participating in the research to post back to the lead researcher. The number of patients recruited will depend on when theoretical saturation is reached (i.e. when the themes relevant to the research have been thoroughly investigated); however it is estimated that approximately 30 patients will be interviewed in total.

Data collection and analysis
Patients who agree to take part in the interviews will be able to choose the location of the interview, either in their own home, at one of the two participating hospitals, or the University of Bristol. Should any participants request to be interviewed at their GP surgery instead, permission will be sought by the chief investigator from the surgery's practice manager to conduct the interview at the surgery. Any travel costs will be reimbursed. Consent will be obtained face-to-face prior to the interview beginning. Should any participants request to be interviewed via telephone, participants will be mailed two copies of the consent form and asked to sign and return them to the chief investigator who will then sign them and return one copy to the participant prior to the interview. An interview topic guide will be applied flexibly to guide interviews. The following broad themes will be explored in interviews: 1) Motivations to undergo surgery; 2) Expected outcomes of surgery; 3) Actual outcomes of surgery (post-op group only); 4) Expectations of follow-up care; 5) Actual experiences of follow-up care (post-op group only). It is anticipated that interviews will last approximately 60 minutes, including 10 minutes dedicated to recording socio-demographic and relevant clinical data at the end of the interview. Interviews will be audio-recorded and transcribed in full. Separate consent will be sought to potentially re-contact them in the future for a follow-up interview.

Coulman KD, *et al. BMJ Open* 2020; 10:e035013. doi: 10.1136/bmjopen-2019-035013

Interviews will be analysed thematically using a grounded theory approach[18]. Data will be analysed using descriptive and explanatory coding to explore and inter-relate categories arising in the data. Data management will be facilitated using the program NVivo 9[19]. Descriptive accounts will be written up relating to each batch of interviews, and matrices will be drawn up to compare the occurrence of themes across interviews. Data analysis will run in parallel with data collection so that emerging themes can be followed up to enrich subsequent interviews. A small sample of the interview transcripts will be independently coded by both the chief investigator and her primary supervisor. The coding structures will then be discussed in a supervision meeting and revised as appropriate. The analysis will also be reviewed with the chief investigator's co-supervisor, and patient research partners.

Findings from the qualitative interviews will be combined with findings from a previous review of qualitative literature in this area, and written up for publication as soon as possible. Particular attention will be paid to ensuring that recommendations relating to the improvement of follow-up care are disseminated as soon as possible to clinicians and policymakers.

Important outcomes identified by patients in the semi-structured interviews will be added to the outcomes previously identified from systematic literature reviews undertaken by the lead researcher to create a comprehensive 'long' list of outcomes important to obesity surgery patients. The list will be reviewed by the lead researcher, her supervisors, and patient research partners to make sure it is comprehensive such that potentially important outcomes have not been omitted. This will form the basis of the next stage of the research (Delphi process).

Delphi process

This comprehensive list of outcomes will be refined into a 'core' outcome set using a Delphi process consisting of two questionnaire rounds where health professionals and patients individually rate each outcome for its value of being included in the core outcome set, followed by consensus meetings to discuss any potential areas of disagreement with regard to which outcomes to include and to ratify the final core outcome set.

Ethical issues arising

Informed consent

Written, informed consent will be obtained from all participants prior to the qualitative interviews. Confidentiality and anonymity will be assured at this stage and the purposes and possible uses of the research will be explained. It will be made clear that participants are free to drop out or delay participation in research at this stage. Separate consent will be sought to digitally-record interviews, and to potentially re-contact the participants in future for a follow-up interview.

Data protection

The principles of the Data Protection Act (1998) will be complied with and data obtained from interviewees will be anonymised using unique study codes. Documents to interpret the codes and personal data will be stored in separate encrypted files in separate locations on the University of Bristol server. All hard copy study documents will be stored in locked filing cabinets. Only data necessary to the purposes of the research will be obtained and stored.

Study Protocol version 2.0 (7/7/14)

Confidentiality

Confidentiality and anonymity outside of the interviews will be discussed and assured prior to beginning each interview. It will also be assured through the processes of data storage outlined above and the use of only fully anonymised quotations in all presentations of research findings.

Protection from distress

It is understood that living with morbid obesity and making the decision to undergo obesity surgery are personal and sensitive issues for patients. Therefore interviews will be undertaken sensitively and patients will only be encouraged to talk about aspects of their illness and care that they feel comfortable with. In the event that a participant becomes distressed during a research interview, the researcher will offer to take a short break from the interview and will remind participants that they do not have to discuss any topics they are uncomfortable with and are free to withdraw their consent to participate in the research at any time. If any patients express particular distress relating to their condition or treatment, a distress protocol will be followed (Appendix 1). The interviewer previously worked as a health professional in a NHS obesity surgery service and has experience of dealing with sensitive issues that may be brought up by this patient population. In addition, the interviewer will be supervised by a trained qualitative researcher who has experience in interviewing morbidly obese patients.

Integrity of research data

The MRC principles of Good Research Practice will be adopted with all primary data being retained so that there is an auditable trail from results back to data. Results will be fully and accurately reported at the end of the project.

Independence and impartiality

Every attempt will be made to maintain reflexivity at every stage of the research and to acknowledge the potential impact of the researcher upon the findings.

Study Protocol version 2.0 (7/7/14)

Appendix 1: Distress protocol - Interviews

All interviews will be prefaced with a statement about confidentiality and the duty of care. Participants will be told that interview is strictly confidential but should they disclose information to suggest that they are at significant risk of harm the researcher *may* have to discuss this with a clinical advisor.

In the event that a participant appears to be distressed during the interview (eg. becomes silent, cries) or discloses information to provoke concern about suicide risk, the following procedures will be followed:

- Participants will be offered the opportunity to pause for a break from the interview and will then be asked if they would like to resume.
- If necessary, the interview will be terminated and recording equipment stopped.
- At first, the interviewer will listen to the interviewee and offer support in situ. This will allow the researcher to assess whether further action is necessary.
- Should the interviewer remain concerned, they will reflect this to the interviewee and depending on the nature of the situation:
    1. Offer information about local help services
    2. Ask the interviewee if there is anyone they should contact, and if so attempt to make contact
    3. Offer to make initial contact with clinical services (primary or secondary) on behalf of the individual and with their consent
- In cases of particular concern the interviewer will
    1. If necessary, remain with the person until their distress has subsided or someone else is present
    2. Contact a local study clinician for advice/ assistance
    3. Provide a written report of the incident to AOS/JB (PhD supervisors), including information about the nature of the distress and the actions taken

Interviewees will be advised to contact their GP should they find subsequently that the interview provokes issues that they need to discuss.

Study Protocol version 2.0 (7/7/14)

Reference List

(1) The NHS Information Centre, Lifestyles Statistics. Statistics on obesity, physical activity and diet: England, 2012. The Health and Social Care Information Centre.

(2) World Health Organization. Fact sheet No. 311: Obesity and overweight. 2006.

(3) Must A, Spadano J, Coakley EH, Field AE, Colditz G, Dietz WH. The disease burden associated with overweight and obesity. *JAMA* 1999; 282(16):1523-1529.

(4) Buchwald H, Oien DM. Metabolic/bariatric surgery Worldwide 2008. *Obesity Surgery* 2009; 19(12):1605-1611.

(5) Picot J, Jones J, Colquitt JL, Gospodarevskaya E, Loveman E, Baxter L et al. The clinical effectiveness and cost-effectiveness of bariatric (weight loss) surgery for obesity: a systematic review and economic evaluation. *Health Technology Assessment* 2009; 13(41):1-+.

(6) U.S.Department of Health and Human Services FDA Center for Drug Evaluation and Research, U.S.Department of Health and Human Services FDA Center for Biologics Evaluation and Research, U.S.Department of Health and Human Services FDA Center for Devices and Radiological Health. Guidance for industry: patient-reported outcome measures: use in medical product development to support labeling claims: draft guidance. *Health & Quality of Life Outcomes* 2006; 4:79.

(7) Lasch KE, Marquis P, Vigneux M, Abetz L, Arnould B, Bayliss M et al. PRO development: rigorous qualitative research as the crucial foundation. *Quality of Life Research* 2010; 19(8):1087-1096.

(8) Coulman KD, Abdelrahman T, Owen-Smith A, Andrews RC, Welbourn CR, Blazeby JM. Measuring patient reported outcomes in bariatric surgery: standards of reporting and synthesis with clinical data. *Br J Surg* 2012; 99(Suppl. 2):1.

(9) University of Liverpool. COMET Initiative: http://www.comet-initiative.org/. 2012.

(10) Medical Research Council. MRC Hubs for Trial Methodology Research: http://www.methodologyhubs.mrc.ac.uk/. 2012.

(11) Tugwell P, Boers M, Brooks P, Simon L, Strand V, Idzerda L. OMERACT: An international initiative to improve outcome measurement in rheumatology. *Trials* 2007; 8.

(12) Hewlett SA. Patients and clinicians have different perspectives on outcomes in arthritis. *Journal of Rheumatology* 2003; 30(4):877-879.

(13) Myers VH, Adams CE, Barbera BL, Brantley PJ. Medical and Psychosocial Outcomes of Laparoscopic Roux-en-Y Gastric Bypass: Cross-sectional Findings at 4-Year Follow-up. *Obesity Surgery* 2012; 22(2):230-239.

(14) American Society for Metabolic & Bariatric Surgery. ASMBS Position Statement on Global Bariatric Healthcare. 2011.

Study Protocol version 2.0 (7/7/14)

(15) Heber D, Greenway FL, Kaplan LM, Livingston E, Salvador J, Still C. Endocrine and Nutritional Management of the Post-Bariatric Surgery Patient: An Endocrine Society Clinical Practice Guideline. *Journal of Clinical Endocrinology & Metabolism* 2010; 95(11):4823-4843.

(16) Ogden J, Clementi C, Aylwin S. The impact of obesity surgery and the paradox of control: A qualitative study. *Psychology & Health* 2006; 21(2):273-293.

(17) Ogden J, Clementi C, Aylwin S, Patel A. Exploring the impact of obesity surgery on patients' health status: a quantitative and qualitative study. *Obesity Surgery* 2005; 15(2):266-272.

(18) Glaser B, Strauss A. The discovery of grounded theory: Strategies for qualitative research. Chicago: Aldine; 1967.

(19) NVivo 9, QSR International; 2010.
