# Supplementary material for: Patients’ experiences of life after bariatric surgery and follow-up care: a qualitative study
Source: BMJ Open. 2020 Feb 6;10(2):e035013. doi: 10.1136/bmjopen-2019-035013 (PMC7045271; doi:10.1136/bmjopen-2019-035013)
Supplement: Supplementary data [file bmjopen-2019-035013supp002.pdf]

Document S2: Topic guide

Post-operative patient interviews

NB This guide is necessarily provisional, as its application will depend on the experience of individual participants.

Introduction

❖ *Researcher explains research, asks if any queries on PIS, and takes consent.*

Motivations to undergo surgery

- To start off, I was just wondering if you could tell me a bit about what factors led to your decision to go for surgery?

Expected outcomes of surgery

- If you can think back to a few weeks before you had the surgery, can you remember how you were feeling about having surgery?
  Probe: Was there anything you were worried about? [Prompt on risks/side-effects of surgery]
  Probe: Was there anything you hoped would change for the better by having the surgery?
  Probe: Was there anything you were hoping to prevent by having surgery?

Actual outcomes of surgery

- How do you feel about having had surgery now?
  Probe: Which operation did you have?
  Probe: How long ago was it now?
  Probe: What's happened since? [Prompt on the things they said they were worried about/hoped would change/hoped they would prevent by having surgery]
  Probe: Was there anything that happened that you didn't expect? [Prompt on during surgery and after surgery]
  Probe: Are you glad you had the surgery?
  Probe: Do you think having the operation has changed your relationships with people who are important to you?
- To you, what are the most important results of surgery?
  If they say weight loss, Probe: What does weight loss mean for you?
  Probe: Are these things different to what you would have said before having surgery?
  Probe: What results of surgery might be most important to other people undergoing the surgery?
  If yes, Probe: How are they different?
- Are there any things that you didn't know beforehand that you wished you had?
- What do you think will happen in the future?

Expectations of follow-up care

- If you can think back to a few weeks before you had the surgery, can you remember if you had any expectations or ideas about the care you would receive after surgery? *[Prompt on short-term inpatient care and long-term care]*

Actual experiences of follow-up care

- Since you've had your surgery, have you been back to the hospital? *[Prompt on the surgical and weight management teams,]*
  Probe: What about your GP practice, have you seen anyone there since your surgery?
  Probe: Do you think there is a role for more follow-up care from primary care?
- What do you think will happen next?
- How do you feel about the NHS care you have received since your surgery?
  Probe: Tell me about the things you found most helpful
  Probe: Tell me about the things you found least helpful
  Probe: Is there anything you would have preferred to have been done differently?/Is there anything you think the NHS could have done to better support you since you've had your surgery?
- Could you say what you thought the most important things of good care after weight loss surgery were?
  Probe: Who would be the best person/people to do that?
  Probe: What would be the best setting for this? [Prompt on hospital/ another setting]
  Probe: Do you think these are the same things that others who've had surgery would say?
- What about support groups – have you been involved with any of these?
  If yes Probe: Do you tend to go to meetings or do you use online forums?
  Probe: How do you find these? [Prompt on positive and negative aspects]
  If no Probe: What's stopped you from getting involved?[ie. prompt on if unaware of them, none convenient, don't find helpful]
  If used to be involved but now no longer involved Probe: What's led to this?

Version 1.2 (18/3/2013)

**Clarifications, etc.**<br>❖ *Researcher asks participant to provide more information where points of interest were not previously probed.*
**Wind-down**<br>❖ *Researcher checks topic guide for omissions and prompts informant back towards areas of discussion where they seemed most comfortable.*
**Case report form**<br>❖ *Researcher records relevant socio-demographic and clinical details using the case report form.*
**Closing**<br>❖ *Researcher asks if patient would be interested in being contacted about a possible follow-up interview – to be recorded on consent form*<br>❖ *Researcher thanks interviewee for participating and asks if they would like a summary of the study results once complete*

Version 1.2 (18/3/2013)
