# Supplementary material for: Patients’ experiences of life after bariatric surgery and follow-up care: a qualitative study
Source: BMJ Open. 2020 Feb 6;10(2):e035013. doi: 10.1136/bmjopen-2019-035013 (PMC7045271; doi:10.1136/bmjopen-2019-035013)
Supplement: Supplementary data [file bmjopen-2019-035013supp003.pdf]

Document S3: Case Report Form - Patient views about weight loss surgery

Participant identification number:<br>Date of birth: __/__/____<br>Initials:<br>Today's date: __/__/____	*If patient has consented to GP being informed about participation in study*:<br>GP name:<br>GP surgery and address:	
1.	What is the gender of the participant? 1=Male, 2=Female	☐
2.	What is the patient's ethnicity? 1=White British, 2=White other, 3=Black, 4=Asian, 5=Mixed, 6=Chinese, 7=Other	☐
3.	Who does the participant live with? 1=alone, 2=with family, 3=with other adults	☐
4.	What is the participant's marital status? 1=single, 2=married, 3=separated, 4=divorced, 5=widowed	☐
5.	What is the highest level of education completed? 1=less than compulsory school education, 2=compulsory school education, 3=post-compulsory school education below university level, eg. advanced technical school/advanced vocational, 4=university level	☐
6.	What is the employment status of the participant? 1=employed full-time (could be on sick leave), 2=employed part-time, 3=homemaker, 4=student, 5=unemployed, 6=retired, 7=self-employed, 8=other (specify)..	☐
7.	What is the current or last occupation of the participant?...	
8a.	What is the patient's surgical status? 1=Awaiting surgery, 2=Undergone surgery (go to 8e.)	☐
b.	If the participant is awaiting surgery, what operation are they hoping to undergo? 1=LAGB, 2=RYGB, 3=SG, 4=Other (specify)..	☐
c.	If the participant is awaiting surgery, do they have a date? 1=Yes, 2=No	☐
d.	If yes, what is the date?	__/__/____
e.	If the participant has undergone surgery, what operation have they undergone? 1=LAGB, 2=RYGB, 3=SG, 4=Other (specify)..	☐
f.	If the participant has undergone surgery, what date did it occur?	__/__/____
9a.	What was/is the participant's weight prior to surgery (self-reported)?	☐ Stones ☐ lbs ☐ Kg
b.	If the participant has undergone surgery, what is their weight now (self-reported)?	☐ Stones ☐ lbs ☐ Kg
10.	What is the participant's height (self-reported)?	☐ feet ☐ inches ☐ cm

Case Report Form (Interviews), Version 1.2 (8/11/2012)

11.	Does the participant have any co-morbidities? List as many as needed. 1=diabetes, 2=hypertension, 3=hyperlipidaemia, 4=cardiac disease (excluding 2 and 3), 5=sleep apnoea, 6=asthma, 7=joint problems (eg. arthritis), 8=urinary incontinence, 9=infertility, 10=other, 11=None. If other, please specify..	☐ ☐<br>☐ ☐

Case Report Form (Interviews), Version 1.2 (8/11/2012)
