# Supplementary material for: Patients’ experiences of life after bariatric surgery and follow-up care: a qualitative study
Source: BMJ Open. 2020 Feb 6;10(2):e035013. doi: 10.1136/bmjopen-2019-035013 (PMC7045271; doi:10.1136/bmjopen-2019-035013)
Supplement: Supplementary data [file bmjopen-2019-035013supp004.pdf]

Document S4: Final coding structure used in NVivo

Name of code	Code related to
General NHS comments not related to surgery	Aspects of care
Guidelines from health professionals	Aspects of care
Follow-up care	Aspects of care
Factors leading to decision to have surgery	Life pre-surgery
Past struggles with weight	Life pre-surgery
Pre-op feelings about surgery	Life pre-surgery
Expectations of surgery	Life pre-surgery
Pre-surgery preparation	Life pre-surgery
Liver shrinking pre-op diet	Life pre-surgery
Other people's perceptions of obesity	Life pre-surgery
NHS funding of surgery	Life pre-surgery
Criteria to fulfill to obtain surgery	Life pre-surgery
Background	Life pre-surgery
Choice of operation	Life pre-surgery
Social impact of obesity	Life pre-surgery
Treatment from health professionals around obesity	Life pre-surgery
Other people's experiences of surgery	Life post-surgery
Future expectations and hopes	Life post-surgery
Peri-operative experience	Life post-surgery
Factors that help maintain motivation post-surgery	Life post-surgery
Support groups	Life post-surgery
Outcome - Activity, mobility	Life post-surgery
Outcome - Body image	Life post-surgery
Outcome - Comorbidity	Life post-surgery
Outcome - Eating	Life post-surgery
6 week post-op diet	Life post-surgery
Outcome - GI symptoms	Life post-surgery
Bowel movements	Life post-surgery
Wind or gas	Life post-surgery
Dumping or sickness	Life post-surgery
Outcome - Hunger	Life post-surgery
Outcome - Psychological	Life post-surgery
Outcome - Social impact of surgery	Life post-surgery
Family reactions to surgery	Life post-surgery
Outcome - Weight	Life post-surgery
In-hospital side-effects of surgery	Life post-surgery
Post-discharge side-effects of surgery	Life post-surgery
Most important outcomes of surgery	Life post-surgery
Outcome - Normality	Life post-surgery
Outcome - Clothing	Life post-surgery
Outcome - increased work options	Life post-surgery
