# Supplementary material for: Patients’ experiences of life after bariatric surgery and follow-up care: a qualitative study
Source: BMJ Open. 2020 Feb 6;10(2):e035013. doi: 10.1136/bmjopen-2019-035013 (PMC7045271; doi:10.1136/bmjopen-2019-035013)
Supplement: Author's manuscript [file bmjopen-2019-035013.draft_revisions.pdf]

BMJ Open is committed to open peer review. As part of this commitment we make the peer review history of every article we publish publicly available.

When an article is published we post the peer reviewers' comments and the authors' responses online. We also post the versions of the paper that were used during peer review. These are the versions that the peer review comments apply to.

The versions of the paper that follow are the versions that were submitted during the peer review process. They are not the versions of record or the final published versions. They should not be cited or distributed as the published version of this manuscript.

BMJ Open is an open access journal and the full, final, typeset and author-corrected version of record of the manuscript is available on our site with no access controls, subscription charges or pay-per-view fees (http://bmjopen.bmj.com).

If you have any questions on BMJ Open's open peer review process please email info.bmjopen@bmj.com

BMJ Open

Patients' experiences of life after bariatric surgery and follow-up care: A qualitative study.

Journal:	*BMJ Open*
Manuscript ID	bmjopen-2019-035013
Article Type:	Original research
Date Submitted by the Author:	15-Oct-2019
Complete List of Authors:	Coulman , Karen ; University of Bristol, Population Health Sciences, Bristol Medical School<br>MacKichan, Fiona; University of Bristol, Population Health Sciences, Bristol Medical School<br>Blazeby, Jane; University of Bristol, Population Health Sciences, Bristol Medical School; University Hospitals Bristol NHS Foundation Trust, Division of Surgery, Head and Neck<br>Donovan, Jenny; University of Bristol, Population Health Sciences, Bristol Medical School; University Hospitals Bristol NHS Foundation Trust, NIHR CLAHRC West<br>Owen-Smith, Amanda; University of Bristol, Population Health Sciences, Bristol Medical School
Keywords:	Organisation of health services < HEALTH SERVICES ADMINISTRATION & MANAGEMENT, Quality in health care < HEALTH SERVICES ADMINISTRATION & MANAGEMENT, QUALITATIVE RESEARCH, Adult surgery < SURGERY

SCHOLARONE™
Manuscripts

I, the Submitting Author has the right to grant and does grant on behalf of all authors of the Work (as defined in the below author licence), an exclusive licence and/or a non-exclusive licence for contributions from authors who are: i) UK Crown employees; ii) where BMJ has agreed a CC-BY licence shall apply, and/or iii) in accordance with the terms applicable for US Federal Government officers or employees acting as part of their official duties; on a worldwide, perpetual, irrevocable, royalty-free basis to BMJ Publishing Group Ltd ("BMJ") its licensees and where the relevant Journal is co-owned by BMJ to the co-owners of the Journal, to publish the Work in this journal and any other BMJ products and to exploit all rights, as set out in our licence.

The Submitting Author accepts and understands that any supply made under these terms is made by BMJ to the Submitting Author unless you are acting as an employee on behalf of your employer or a postgraduate student of an affiliated institution which is paying any applicable article publishing charge ("APC") for Open Access articles. Where the Submitting Author wishes to make the Work available on an Open Access basis (and intends to pay the relevant APC), the terms of reuse of such Open Access shall be governed by a Creative Commons licence – details of these licences and which Creative Commons licence will apply to this Work are set out in our licence referred to above.

Other than as permitted in any relevant BMJ Author's Self Archiving Policies, I confirm this Work has not been accepted for publication elsewhere, is not being considered for publication elsewhere and does not duplicate material already published. I confirm all authors consent to publication of this Work and authorise the granting of this licence.

For peer review only

Title: Patients' experiences of life after bariatric surgery and follow-up care: A qualitative study.

Authors: Karen D Coulman (karen.coulman@bristol.ac.uk)[1], Fiona MacKichan (fiona.mackichan@bristol.ac.uk)[1], Jane M Blazeby (j.m.blazeby@bristol.ac.uk)[1,2], Jenny L Donovan (jenny.donovan@bristol.ac.uk)[1,3], Amanda Owen-Smith (a.owen-smith@bristol.ac.uk)[1]

Affiliations: [1]Population Health Sciences, Bristol Medical School, University of Bristol, Bristol, UK

[2]Division of Surgery, Head and Neck, University Hospitals Bristol NHS Foundation Trust, Bristol, UK

[3]NIHR CLAHRC West, University Hospitals Bristol NHS Foundation Trust, Bristol, UK

Keywords: Adult surgery, Qualitative Research, Organisation of health services, Quality in health care

Running Title: Bariatric surgery and follow-up care

Corresponding author: Dr Karen Coulman, Population Health Sciences, Bristol Medical School, University of Bristol, 1-5 Whiteladies Road, Bristol, BS8 1NU, UK. Email: karen.coulman@bristol.ac.uk, Telephone: +44/0 117 42 83132.

Word count (main body of text excluding abstract, article summary, acknowledgements, references and tables): 4300

Funding: This article summarizes independent research funded by a National Institute for Health Research (NIHR) Doctoral Research Fellowship (KD Coulman, DRF-2011-04-032). This work was also supported by the Medical Research Council (MRC) ConDuCT-II Hub (Collaboration and innovation for Difficult and Complex randomised controlled Trials In Invasive procedures - MR/K025643/1). The views and opinions expressed in this publication are those of the authors and do not necessarily reflect those of the National Health Service, the NIHR the Department of Health, or the MRC. JMB is an NIHR Senior Investigator.

Competing interests: None declared.

License statement: I, the Submitting Author has the right to grant and does grant on behalf of all authors of the Work (as defined in the below author licence), an exclusive licence and/or a non-exclusive licence for contributions from authors who are: i) UK Crown employees; ii) where BMJ has agreed a CC-BY licence shall apply, and/or iii) in accordance with the terms applicable for US Federal Government officers or employees acting as part of their official duties; on a worldwide, perpetual, irrevocable, royalty-free basis to BMJ Publishing Group Ltd ("BMJ") its licensees and where the relevant Journal is co-owned by BMJ to the co-owners of the Journal, to publish the Work in BMJ Open and any other BMJ products and to exploit all rights, as set out in our licence.

The Submitting Author accepts and understands that any supply made under these terms is made by BMJ to the Submitting Author unless you are acting as an employee on behalf of your employer or a postgraduate student of an affiliated institution which is paying any applicable article publishing charge ("APC") for Open Access articles. Where the Submitting Author wishes to make the Work available on an Open Access basis (and intends to pay the relevant APC), the terms of reuse of such Open Access shall be governed by a Creative Commons licence – details of these licences and which Creative Commons licence will apply to this Work are set out in our licence referred to above.

For peer review only - http://bmjopen.bmj.com/site/about/guidelines.xhtml

UOB Confidential

Abstract

Objectives: Bariatric surgery is the most clinically effective treatment for people with severe and complex obesity, however, the psychosocial outcomes are less clear. Follow-up care after bariatric surgery is known to be important, but limited guidance exists on what this should entail, particularly related to psychological and social well-being. Patients' perspectives are valuable to inform the design of follow-up care. This study investigated patients' experiences of life after bariatric surgery including important aspects of follow-up care.

Design: A qualitative study using semi-structured individual interviews. A constant comparative approach was used to code data and identify themes and overarching concepts.

Setting: Bariatric surgery units of two publicly funded hospitals in the South of England.

Participants: Seventeen adults (10 women) that underwent a primary operation for obesity (mean time since surgery 3.11 years), including Roux-en-Y gastric bypass, adjustable gastric band and sleeve gastrectomy, agreed to participate in the interviews.

Results: Experiences of adapting to life following surgery were characterised by the concepts of 'normality' and 'ambivalence', while experiences of 'abandonment' and 'isolation' dominated participants' experiences of follow-up care. Patients highlighted the need for more flexible, longer-term follow-up care that addresses social and psychological difficulties post-surgery and integrates peer support.

Conclusions: This research highlights unmet patient need for more accessible and holistic follow-up care that addresses the long-term multi-dimensional impact of bariatric surgery. Future research should investigate effective and acceptable follow-up care packages for patients undergoing bariatric surgery.

For peer review only - http://bmjopen.bmj.com/site/about/guidelines.xhtml

UOB Confidential

Article Summary - strengths and limitations of this study

- Patients who had undergone all three main types of bariatric procedures across two UK centres were included in the research.
- A detailed qualitative approach was used, allowing participants to relate their own experiences in terms that were relevant for them.
- A rigorous approach to analysis was undertaken, including independent coding of initial transcripts by three researchers, and agreement of emergent themes throughout analysis with at least one other qualitative researcher.
- It is not known whether similar themes would be found with participants in other centres.
- Findings relating to follow-up care may be less generalisable to healthcare systems with different service pathways and funding structures.

For peer review only

UOB Confidential

Introduction

Over 650 million or 13% of adults worldwide suffer from obesity (body mass index (BMI) ≥30 kg/m²), representing a tripling of figures since 1975.[1] Obesity is associated with an increased risk of type 2 diabetes, cardiovascular disease, certain cancers, and premature death.[2 3] Within this population, people with severe and complex obesity (BMI ≥40 kg/m2, or 35-40 kg/m2 with another significant health problem that could be improved by weight loss) suffer the greatest health burdens and are at the highest risk of premature death.[4 5] In addition to the physical and metabolic health burdens, people with severe and complex obesity are more likely to suffer with psychological disorders such as depression, anxiety and disordered eating, and reduced health-related quality of life (HRQL).[6 7] These individuals also suffer from social stigma and discrimination related to their weight [6 8] which is in turn associated with adverse physical and psychological outcomes.[8 9] Thus, any interventions to treat severe and complex obesity should consider the impact on these psychosocial outcomes in addition to traditional clinical and metabolic outcomes.[10 11]

Bariatric surgery, combined with behaviour change and dietary management, is the most clinically effective treatment for people with severe and complex obesity, in terms of weight loss and the improvement of co-morbidities such as type 2 diabetes.[5 12 13] However the impact of bariatric surgery on HRQL is less clear, with some aspects of HRQL improving but others not.[11 12 14] Previous qualitative research has highlighted the complex and changeable nature of the psychosocial impact of bariatric surgery, helping to shed light on some of these inconsistencies in the HRQL literature, and emphasising the importance of long-term post-operative support in helping patients manage these changes.[10 15] Previous research has also reported attendance at follow-up visits to be associated with better weight loss outcomes after bariatric surgery.[16]

Follow-up care is thus important to optimise clinical and psychosocial outcomes of bariatric surgery. However, bariatric surgery follow-up care has been reported to vary greatly across the UK,[17] and

For peer review only - http://bmjopen.bmj.com/site/about/guidelines.xhtml

UOB Confidential

current UK and US bariatric surgery guidelines focus on surgical and metabolic outcomes, with limited guidance on how to support psychological, social and lifestyle changes that affect patients' HRQL.[5 18] Nevertheless, previous work has highlighted the importance of these multi-faceted aspects of HRQL to patients that have undergone bariatric surgery and recommendations are needed on how best to support patients after surgery to optimise these outcomes.[19 20]

In seeking to evaluate and provide recommendations on bariatric surgery follow-up care, the patient's perspective can provide valuable information.[21] Qualitative research is useful to explore patients' perspectives as it seeks to gain the insider's view on how people view, experience, and make sense of their social world.[22-24] Previous qualitative studies have identified patient need for longer follow-up after bariatric surgery, better access to psychological support, and access to health professional advice between routine appointments.[15 25-32] However, these studies are limited in that most are single-centre and report findings from select groups, for example patients that had undergone one type of bariatric procedure only (e.g. adjustable gastric band) or those that had experienced negative outcomes such as weight re-gain or substance abuse issues. There is a need to understand the most important aspects of bariatric surgery follow-up care from the perspectives of a range of patients that have undergone different bariatric procedures and at different timepoints post-surgery to provide recommendations for follow-up care. This is the objective of the study presented here.

Methods

Patients who had undergone a primary operation for obesity at two publicly funded bariatric surgery centres in the South of England were eligible to participate in the research. Patients were identified by health professionals at each hospital using databases and clinic lists and sent information about the research. Interested patients contacted the researcher directly (KDC). For initial interviews, patients were sampled purposively, aiming for maximum variation in gender, age, starting body

For peer review only - http://bmjopen.bmj.com/site/about/guidelines.xhtml

UOB Confidential

mass index (BMI), type of operation, and time since operation. Emerging findings from analysis of initial interviews guided sampling for remaining interviews.[33] Sampling continued until themes were well-established with few or no new insights gained from additional data collection.[23 33] Ethical approval for the study was obtained from Northwest - Preston Research Ethics Committee (Ref 12/NW/0844). This study was undertaken as part of a wider study to develop a core outcome set for bariatric surgery (see **document S1** for protocol).[19 20]

Data were collected through semi-structured interviews, mostly conducted in participants' homes. Written informed consent was taken and interviews conducted according to an outline topic guide, which evolved iteratively as the research progressed (see **document S2** for final versions). Relevant demographic and clinical information were also collected (**document S3**). All interviews were conducted and audio-recorded between February 2013 and November 2014, by a female researcher (KDC) who was a PhD student and registered dietitian. KDC underwent training in qualitative research methods and was supervised by two experienced qualitative researchers (AOS, FM). An initial telephone conversation was held with each participant to discuss the study and arrange the interview. Participants were otherwise not previously known to the researcher prior to interview. The researcher introduced herself as a PhD student to participants. She did not reveal her professional background as a registered dietitian unprompted but did not seek to hide it if participants asked. Field notes, which provided important contextual information to aid data analysis, were made as soon as possible after each interview.[34]

Recorded interviews were transcribed verbatim, and transcriptions checked for accuracy by KDC. Thematic analysis was undertaken, using techniques of constant comparison to code data and identify emerging themes.[33] Coding was completed for all transcripts by KDC, with a sample of transcripts independently coded by two other experienced qualitative researchers (AOS and JLD) (see **document S4** for final coding framework). Differences in interpretation were resolved through discussion. Coding and data management were facilitated using NVivo 10 software.[35] Detailed

For peer review only - http://bmjopen.bmj.com/site/about/guidelines.xhtml

UOB Confidential

descriptive accounts were written by KDC for each small batch of interviews, whereby emergent themes were identified and further investigated in subsequent interviews. Finally, large matrices were created to compare themes across all participants and identify concepts overarching all themes.[36] AOS, FM, JLD and JMB reviewed all descriptive accounts and made suggestions about further links between themes and concepts.

Two patient research partners who had themselves undergone NHS-funded bariatric surgery reviewed and provided feedback on the research proposal, the interview topic guide, and all written patient information (including study recruitment documents, and the final study summary disseminated to participants).

Results

Of 48 patients invited, 17 agreed to take part in interviews (mean time since surgery 3.11 years), although two others (spouses of existing participants) were opportunistically recruited as the research was ongoing. Interviews lasted between 44 and 110 minutes. Twelve of the 19 participants were female, and the mean age was 51.1 years. All reported their ethnicity to be ‘White British’, and 17 had already undergone surgery **(Table 1)**. The analysis presented draws on interview data from the 17 participants that had undergone surgery.

Bariatric surgery was a life-changing journey for participants, impacting on several different areas of their lives. The overarching concepts of ‘normality’ and ‘ambivalence’ emerged from analysis of data on patients’ experiences of adapting to life after surgery. Analysis of data relating to experiences of follow-up care was conducted separately and characterised by two concepts – ‘abandonment’ and ‘isolation’. Results are presented according to overarching concept with participant quotes used to support the description of each concept.

For peer review only - http://bmjopen.bmj.com/site/about/guidelines.xhtml

UOB Confidential

For peer review only

Adapting to life after surgery – normality and ambivalence

Throughout several areas of their lives, participants were striving to be more "normal" after bariatric surgery. This related to their physical and psychological health, eating patterns, weight, and social functioning. Participants experienced many positive changes that undeniably brought them closer to their idea of normality. However, participants also described things that did not change, for which they still felt abnormal. Some also experienced changes perceived as negative or difficult to deal with, which made them feel more abnormal and required a process of adjustment. This was acknowledged as a "trade-off", or the "price to pay" (P08) for the benefits gained. The complexity of the changes experienced highlighted the ambivalence of living with the results of bariatric surgery. Despite the challenges, all participants felt the surgery was a good decision: "I don't regret it for a minute. Despite all the complications and issues." (P14)

Normality

All participants reported an improvement in activity and mobility levels and/or their ability to carry out 'normal' activities of daily living following surgery: "I'm more mobile, I can tie my shoelaces, shower properly…my life has changed for the better." (P10). Participants also reported several positive changes related to physical and psychological health including a reduction in medications required (e.g. for diabetes), an improvement in physical symptoms (such as joint pain), self-confidence, and psychological well-being: "I feel healthier mentally in my head, like I want to get out there." (P09).

Some participants described an improved or more 'normal' relationship with food after surgery, whereby they had retrained their mind to focus on "eating more sensibly" rather than thinking they were "on a diet" (P11). Others experienced no real change to their relationship with food, feeling as though they still had to be "on a permanent diet" (P19), or continued to use food as way of coping with difficult emotions which remained: "I still have an awkward relationship with food…still have the same demons…I probably rely on food to deal with certain emotions" (P14).

For peer review only - http://bmjopen.bmj.com/site/about/guidelines.xhtml

UOB Confidential

For peer review only

All 17 of the participants had lost a large amount of weight since having surgery, however, eight had re-gained some of this weight. Participants reported feeling distressed by this as they did not want to return to the way they were: “That was a real horrendous thing for me to see my weight go up a bit after all I’d gone through to get it down...” (P07). However, a couple described being reassured by health professionals that it was normal to experience some weight re-gain. The majority related their weight re-gain to a gradual increase in appetite and/or portion sizes over time (which had initially decreased after surgery), and a feeling that the surgery was not as effective as it had been: “I don’t seem to be getting the urge to stop quicker, like I did before” (P18).

The majority of participants reported developing loose-hanging excess skin following their massive weight loss, which challenged their sense of normality. Although they were pleased to be a more ‘normal’ size, some felt ashamed of how abnormal their body looked without clothes on. Skin removal surgery was a costly option, so some had learned to live with the excess skin; however, a few found the excess skin to be particularly problematic, impacting on their mental health and relationships: “My husband doesn’t like the excess skin…and that’s one of the reasons why I must do something about it, because…I know I look like a bag of s**t” (P12).

Ambivalence

Although improvements to existing health problems were important benefits of the surgery, five participants reported developing new health problems post-surgery, including micronutrient deficiencies, menstrual problems, brittle bones, low blood pressure and cardiac issues: “…you give up one set of health implications but you get given another set in its place...” (P07). Some participants still suffered with several food intolerances and/or frequent gastrointestinal symptoms many years after surgery, which they reported resulted in a poorly balanced diet: “I can’t eat bread or meat…That’s one of the small prices I have to pay…my intake of food is nowhere near balanced…” (P08).

For peer review only - http://bmjopen.bmj.com/site/about/guidelines.xhtml
UOB Confidential

Difficulties were described in developing new coping strategies to replace food, which had previously been a “comfort blanket”: “…all your insides are different but your brain…no different whatsoever…that for me was the hardest thing to adjust to, because my brain was still telling my stomach I was hungry but obviously I couldn’t [eat]...” (P03). One patient described developing an alcohol dependency post-surgery (which they had eventually overcome), and two participants mentioned the need for more psychological input to help with their adjustment following surgery: “There was no formal counselling…and that might be a good idea to find out why we eat so much, why are we addicted to food…” (P04).

Social functioning and stigma

Ambivalence was also evident in participants’ experiences of social functioning and stigma. Participants reported receiving positive attention due to their weight loss: “…people tell you ‘you look brilliant’…that is the good side of it” (P17), however, for some, this led to mixed emotions at the revelation of “how negative people saw you before” (P07). Others described receiving less negative attention and feeling less socially stigmatised due to their obesity: “I can walk down the road now and not get such the bad looks as I used to.” (P04). However, a number of participants had experienced a new type of social stigma at having taken the “easy way out” (P02) by having surgery (e.g. not achieved weight loss through the ‘normal’ means) or being ashamed to tell others they had undergone surgery for fear of this reaction.

Family relationships were impacted in different ways following surgery. Some participants felt more able to participate in parenting and family life; for example, P09 described being able to “plan things as a family” due to improved health and mobility. However, others experienced marital conflict, with some thinking their partners felt insecure due to their changed appearance and improved confidence: “I think my husband was expecting me to lose weight and…go out and find another man…we went through a very bad…4 or 5 months…” (P03).

For peer review only

UOB Confidential

Although many social interactions improved, social eating situations caused anxiety for some due to attracting attention for only eating very small amounts, or unpleasant and embarrassing gastrointestinal symptoms which could arise when eating. This caused some to avoid social events involving food, and/or eat alone at home, separately to family. For some this had remained an issue several years following surgery causing disruption to relationships: “It disrupts life because I can be eating and whether it’s the wrong food, a mouthful too much…I’ve got to go out and she can hear me retching, and it puts her off her food” (P08). Others were able to adapt or reported their social life had “come back” (P10) gradually as food tolerance improved.

Experiences of follow-up care – abandonment and isolation

Participants explained that follow-up care received after surgery was mainly provided by the specialist bariatric surgery team (although what this entailed was highly variable), with little support from their GPs (general practitioners). Only a few participants described feeling well-supported overall, and all of these had undergone their surgery less than two years previously. However, most described at least one aspect of follow-up care which they found helpful. These included: 1) the routine monitoring of certain measures (e.g. weight, nutritional blood tests); 2) the availability of one key health professional (generally a specialist dietitian or nurse), who was easy to contact on an ad-hoc basis; 3) the ability to contact the bariatric team using a range of contact options (e.g. telephone, email); 4) good communication between team members; and 5) continuity of care (e.g. being able to see the same professionals at every appointment) **(Table 2)**.

Overall, however, there was a sense of abandonment and isolation in participants’ accounts of follow-up care. Participants felt that health professionals didn’t always appreciate the long-term implications of life after surgery, or even if they did, services were not set up to support them adequately: “It happened eight years ago so no one thinks you may have any hang-ups, issues, concerns about it...the implications of the changes it makes people don't really appreciate, it’s an old record, old news.” (P07).

For peer review only - http://bmjopen.bmj.com/site/about/guidelines.xhtml

UOB Confidential

For peer review only

Abandonment

Some participants felt that problems or complications they experienced following surgery were ignored or not dealt with properly, or there was a lack of clarity in terms of who to go to if they experienced problems. P07, for example, felt her post-operative problems were dismissed by the specialist team, and that she "was upsetting someone's figures by having complications". P12 experienced a problem with one of her surgical wounds which wouldn't heal and wasn't sure who to go to about it. She felt "quite abandoned" and dealt with it mainly on her own. Abandonment also related to the feeling they had been given inadequate information or guidance about life following surgery: "They give you loads of information about what to do in the first six weeks and then there's nothing…" (P04).

Most participants also reported feeling abandoned by their GPs who were not usually supportive of them having undergone bariatric surgery and did not "fully appreciate the struggles that you have" (P14) in the long-term. However, a minority of participants described feeling well-supported by their GPs who recognised the long-term health benefits of bariatric surgery: "…with being my dad's doctor, he sees that hopefully I won't have the same problems…he's done everything he can to help me…" (P05).

Isolation

Two participants (P09, P15) commented that although the follow-up care they had received from the specialist team had been very good, they had had to make the effort to ask for it: "I feel that as long as you didn't contact them then you will be left alone…" (P15). Concerns were raised for others whom they perceived less likely to seek help proactively: "…these people aren't coming forward to explain that they're having problems because they don't want to feel like a failure…" (P09). P18 expressed disappointment that he had not been sent any appointments post-operatively and felt he had been left "in limbo" to "get on with it" himself. He had not asked for help and was under the impression that it would only be appropriate to contact the team if you were having complications:

For peer review only - http://bmjopen.bmj.com/site/about/guidelines.xhtml

UOB Confidential

For peer review only

"…obviously if I was in excruciating pain from the operation I suppose, I could have gone back…" (P18).

Several participants did not live locally to the hospital where the specialist team were located. This presented a barrier to accessing follow-up care, which some felt could contribute to feelings of isolation: "From this side of the county it's (hospital) extremely difficult to get to…I can understand an awful lot of people thinking "if I ring [hospital] they're going to say come over and see me and that is so difficult to get to...I won't bother." (P15)

Isolation was also apparent in participants' experiences of bariatric surgery peer support groups. Although not part of medical care, these represented an important source of support. These groups were typically run by patient volunteers, with limited or no input from health professionals. Some participants had access to these groups in their local areas, whereas others did not. Those unable to access a group felt this contributed to their sense of isolation post-surgery: "…there's meetings where you can meet other people who've had the [gastric] band…but there's no local ones for me…if people said, 'If you do eat it, it's going to hurt but it will go, and this is the reason it's hurting,' then I could have dealt with it a little bit better." (P17). Those that had accessed these groups described variable experiences. Some found them supportive, for example P01 who continued to attend several years post-surgery, whereas others had negative experiences and felt quite isolated from other members. P19, for example, described her local group as being very "cliquey" with members using the group mainly to emphasize negative experiences or "how to cheat the band". Many felt that peer support groups should be part of routine clinical care to improve accessibility, and that "a chairman" knowledgeable in the results of bariatric surgery was needed to "control the questions and answer session" (P15) and ensure consistency.

For peer review only - http://bmjopen.bmj.com/site/about/guidelines.xhtml

UOB Confidential

For peer review only

Discussion

This in-depth qualitative study found that bariatric surgery impacted participants' physical and psychological health, eating behaviours, weight, and social functioning. The overarching concepts of normality and ambivalence represented their lived experience following bariatric surgery. Normality was evidenced through participants' relief at feeling more normal in some ways (e.g. improved ability to undertake daily activities), yet feeling less normal in other areas, including the development of excess skin and difficulties eating 'normally' in social situations. Although participants experienced many positive health changes, they also experienced changes which were negative or difficult to adapt to, such as an inability to rely on emotional eating as an entrenched coping mechanism, perceived bodily deformity as a result of excess skin, and the destabilisation of important relationships. This highlights the ambivalence of living with the outcomes of bariatric surgery. In coping with these changes, participants received varying levels of care from specialist health professionals and GPs. Although there were some positive experiences, 'abandonment' and 'isolation' characterised most follow-up care experiences. This included feeling unsupported with post-surgery problems (other than serious complications), lack of guidance with long-term lifestyle changes, lack of understanding from GPs, and limited peer support. However, all participants felt that undergoing the surgery was a good decision despite the difficulties. These findings are important in helping to define future follow-up care packages to better address the complex changes experienced after bariatric surgery.

Our findings are consistent with the results of two recent systematic reviews of qualitative research studies which described the multi-dimensional impact of bariatric surgery on patients' lives and the complexity of patient follow-up care needs.[10 15] However, this study contributed additional insights, including highlighting the individual and complex nature of how bariatric surgery changes people's relationship with food in different ways, and changes over time, indicating the need for individualised/tailored support at different timepoints. The importance placed by participants on

For peer review only - http://bmjopen.bmj.com/site/about/guidelines.xhtml

UOB Confidential

social and family eating, and the difficulties experienced after bariatric surgery was also notable and should be given more attention in follow-up care.

Our study confirms previous qualitative findings on the importance of continuity of care,[15] the ability to access professional advice (often from the specialist dietitian) between appointments via telephone or email,[28] the lack of psychological support,[15 25-27 29 30 37] and the need for moderation in patient support groups accessed by patients.[30 31] Previous studies have related patients' views that GPs were not equipped to adequately support them post-surgery.[15 27 28 38] This was also evident in our study with most participants describing negative experiences with GPs in relation to bariatric surgery, and feeling they were unable to offer adequate support. Despite this, several participants would have preferred to access support locally due to living remotely.

Our study highlighted patients' views that bariatric surgery services were not set up to support them with long-term issues, with their experiences of follow-up care characterised by concepts of 'abandonment' and 'isolation'. These echo the concept of recursivity described in the broader help-seeking literature concerning vulnerable groups.[39 40] Recursivity refers to how an individual's previous experience of health services shapes their future help-seeking behaviour.[39 40] In studies of 'hard to reach' groups with mental health difficulties, reticence to seek help was influenced by participants' previous experiences with health professionals not recognising or being dismissive of their problems, being made to feel their problems were their "own fault", and concerns they would be discriminated against or stigmatised.[39 41 42] Previous research in obese individuals suggests they also may delay or avoid seeking healthcare due to societal and medical stigmas.[43 44] This has also been reported by Throsby who conducted a UK-based ethnographic study within a surgical weight management clinic.[45] She described examples of patients struggling with their eating habits and weight post-surgery, and the shame they felt at doing 'badly' after undergoing publicly-funded surgery. The author argued that this "moral weight" could lead to patients not seeking help when most needed.[45] Similarly, feelings of shame and failure at not having met the perceived post-

For peer review only

For peer review only - http://bmjopen.bmj.com/site/about/guidelines.xhtml

UOB Confidential

operative expectations was one reason cited by Australian patients for non-attendance in bariatric surgery aftercare.[27]

The main strength of this research is that a detailed qualitative approach to data collection was used, whereby participants were given the time and flexibility to relate their own experiences in terms that were relevant for them. A rigorous approach to analysis was undertaken, including independent coding of initial transcripts by three researchers, and discussion and agreement of emergent themes throughout analysis with at least one other qualitative researcher. An additional strength was that patients who had undergone all three main types of bariatric procedures across two UK centres were included in the research; however, it is not known whether similar themes would be found with participants in other centres. The findings relating to follow-up care may be less generalisable to healthcare systems with different service pathways and funding structures.

Taken together with previous literature, our findings highlight that current bariatric surgery follow-up care provision is not often aligned with patient need. Patients highlighted the need for a flexible and long-term approach to follow-up care from a multi-disciplinary health professional team. This includes both routine and open appointments, moderated peer support groups, and different methods of contact (e.g. telephone, online in addition to face-to-face). In addition to individual dietary and psychological support, services should consider how to better support patients in developing strategies to cope with family and social difficulties post-surgery. This may include actively engaging family and close friends in pre-operative preparation and/or post-operative interventions. Future research is needed to define and evaluate an effective and acceptable follow-up care package that could be consistently applied across bariatric surgery centres. This may include the optimal systems or pathways to identify and support those who need the most help but are the least likely to seek it, ways of engaging family and social support, and delivering moderated peer support groups. The relative merits of delivering follow-up care in specialist or community-based health services or how it might be shared between the two should also be investigated.

For peer review only - http://bmjopen.bmj.com/site/about/guidelines.xhtml

UOB Confidential

For peer review only

Author contributions

KDC led the study design, data collection and data analysis as part of her PhD research, and drafted this manuscript. AOS, FM and JMB were KDC's PhD supervisors and advised on study design, data collection and analysis, and provided comments on this manuscript. JLD advised and contributed to data analysis and provided comments on this manuscript. All authors approved the final submitted manuscript.

Acknowledgements

The authors would like to thank the study participants who gave up their time to take part in the research and the health professionals at the centres who helped with study recruitment.

Data sharing

Anonymised participant data can be made available on reasonable request to the corresponding author at karen.coulman@bristol.ac.uk.

For peer review only

UOB Confidential

References

1. World Health Organisation. Obesity and overweight. 16 February 2018. https://www.who.int/en/news-room/fact-sheets/detail/obesity-and-overweight. Date accessed 15.10.19.
2. Must A, Spadano J, Coakley EH, et al. The disease burden associated with overweight and obesity. *JAMA* 1999;282(16):1523-29.
3. Wang Y, McPherson K, Marsh T, et al. Health and economic burden of the projected obesity trends in the USA and the UK. *Lancet* 2011;378(9793):815-25.
4. Dietz WH, Baur LA, Hall K, et al. Management of obesity: improvement of health-care training and systems for prevention and care. *Lancet* 2015;385(9986):2521-33.
5. National Institute for Health and Care Excellence. Obesity: identification, assessment and management. Clinical Guideline [CG189]. London, 2014. Available from: https://www.nice.org.uk/guidance/cg189. Date accessed: 15.10.19
6. Collins J, Meng C, Eng A. Psychological Impact of Severe Obesity. *Current Obes Rep* 2016;5(4):435-40.
7. Kolotkin RL, Crosby RD, Williams GR. Health-related quality of life varies among obese subgroups. *Obes Res* 2002;10(8):748-56.
8. Owen-Smith A, Donovan J, Coast J. "Vicious circles": the development of morbid obesity. *Qual Health Res* 2014;24(9):1212-20.
9. Wu YK, Berry DC. Impact of weight stigma on physiological and psychological health outcomes for overweight and obese adults: A systematic review. *J Adv Nurs* 2018;74(5):1030-42.
10. Coulman KD, MacKichan F, Blazeby JM, et al. Patient experiences of outcomes of bariatric surgery: a systematic review and qualitative synthesis. *Obes Rev* 2017;18(5):547-59.
11. Coulman KD, Abdelrahman T, Owen-Smith A, et al. Patient-reported outcomes in bariatric surgery: a systematic review of standards of reporting. *Obes Rev* 2013;14(9):707-20.
12. Colquitt Jill L, Pickett K, Loveman E, et al. Surgery for weight loss in adults. *Cochrane Database Syst Rev* 2014 Aug 8;(8):CD003641. doi: 10.1002/14651858.CD003641.pub4
13. O'Brien PE, MacDonald L, Anderson M, et al. Long-term outcomes after bariatric surgery: fifteen-year follow-up of adjustable gastric banding and a systematic review of the bariatric surgical literature. *Ann Surg* 2013;257(1):87-94.
14. Lindekilde N, Gladstone BP, Lubeck M, et al. The impact of bariatric surgery on quality of life: a systematic review and meta-analysis. *Obes Rev* 2015;16(8):639-51.
15. Parretti HM, Hughes CA, Jones LL. 'The rollercoaster of follow-up care' after bariatric surgery: a rapid review and qualitative synthesis. *Obes Rev* 2019;20(1):88-107.
16. Spaniolas K, Kasten KR, Celio A, et al. Postoperative Follow-up After Bariatric Surgery: Effect on Weight Loss. *Obes Surg* 2016;26(4):900-3.
17. National Confidential Enquiry into Patient Outcome and Death. Too Lean a Service? A review of the care of patients who underwent bariatric surgery. 2012. https://www.ncepod.org.uk/2012report2/downloads/BS_fullreport.pdf. Date accessed: 15.10.19.
18. Mechanick JI, Youdim A, Jones DB, et al. Clinical Practice Guidelines for the Perioperative Nutritional, Metabolic, and Nonsurgical Support of the Bariatric Surgery Patient—2013 Update: Cosponsored by American Association of Clinical Endocrinologists, The Obesity Society, and American Society for Metabolic & Bariatric Surgery. *Surg Obes Relat Dis* 2013;9(2):159-91.
19. Coulman KD, Howes N, Hopkins J, et al. A Comparison of Health Professionals' and Patients' Views of the Importance of Outcomes of Bariatric Surgery. *Obes Surg* 2016;26(11):2738-46.

For peer review only - http://bmjopen.bmj.com/site/about/guidelines.xhtml

UOB Confidential

20. Coulman KD, Hopkins J, Brookes ST, et al. A Core Outcome Set for the Benefits and Adverse Events of Bariatric and Metabolic Surgery: The BARIACT Project. *PLoS Med* 2016;13(11):e1002187. doi: 10.1371/journal.pmed.1002187 [published Online First: 2016/11/30]
21. Richards T, Montori VM, Godlee F, et al. Let the patient revolution begin. *BMJ* 2013;346:f2614. doi: 10.1136/bmj.f2614
22. 1 Pope C, Mays N. Qualitative Methods in Health Research. In: Pope C, Mays N, eds. Qualitative Research in Health Care, Third edition. Malden, Massachusetts: Blackwell Publishing Ltd 2006:12-20.
23. 9 Carter S, Henderson L. Approaches to qualitative data collection in social science. In: Bowling A, Ebrahim S, eds. Handbook of Health Research Methods: Investigation, Measurement and Analysis. Maidenhead, Berkshire: Open University Press 2005:215-29.
24. 1 Snape D, Spencer L. The Foundations of Qualitative Research. In: Ritchie J, Lewis J, eds. Qualitative Research Practice: A Guide for Social Science Students and Researchers. London: SAGE Publications Ltd 2003:1-23.
25. Ogden J, Avenell S, Ellis G. Negotiating control: Patients' experiences of unsuccessful weight-loss surgery. *Psychol Health* 2011;26(7):949-64.
26. Ivezaj V, Saules KK, Wiedemann AA. "I didn't see this coming.": why are postbariatric patients in substance abuse treatment? Patients' perceptions of etiology and future recommendations. *Obes Surg* 2012;22(8):1308-14.
27. Moroshko I, Brennan L, Warren N, et al. Patients' Perspectives on Laparoscopic Adjustable Gastric Banding (LAGB) Aftercare Attendance: Qualitative Assessment. *Obes Surg* 2014;24(2):266-75.
28. Aarts MA, Sivapalan N, Nikzad SE, et al. Optimizing Bariatric Surgery Multidisciplinary Follow-up: a Focus on Patient-Centered Care. *Obes Surg* 2017;27(3):730-36.
29. Lauti M, Stevenson S, Hill AG, et al. Patient Perspectives About Follow-Up Care and Weight Regain Following Sleeve Gastrectomy. *Obes Surg* 2016;26(11):2724-31.
30. Sharman M, Hensher M, Wilkinson S, et al. What are the support experiences and needs of patients who have received bariatric surgery? *Health Expect* 2017;20(1):35-46.
31. Jones L, Cleator J, Yorke J. Maintaining weight loss after bariatric surgery: when the spectator role is no longer enough. *Clin Obes* 2016;6(4):249-58.
32. Pfeil M. Patients' perception of the nursing contribution to gastric band surgery. *Br J Nurs* 2014;23(2):95-9.
33. Glaser B, Strauss A. The discovery of grounded theory: Strategies for qualitative research. Chicago: Aldine 1967.
34. 6 Legard R, Keegan J, Ward K. In-depth Interviews. In: Ritchie J, Lewis J, eds. Qualitative Research Practice: A Guide for Social Science Students and Researchers. London: SAGE Publications Ltd 2003:138-69.
35. NVivo 10. Melbourne: QSR International Pty Ltd., 2013.
36. Strauss A, Corbin J. Basics of qualitative research: Grounded theory procedures and techniques. Newbury Park, CA: SAGE 1990.
37. Stolzenberger KM, Meaney CA, Marteka P, et al. Long-term quality of life following bariatric surgery: A descriptive study. *Bariatr Surg Pract Patient Care* 2013;8(1):29-38.
38. Jose K, Venn A, Nelson M, et al. A qualitative study of the role of Australian general practitioners in the surgical management of obesity. *Clin Obes* 2017;7(4):231-38.
39. Bristow K, Edwards S, Funnel E, et al. Help Seeking and Access to Primary Care for People from "Hard-to-Reach" Groups with Common Mental Health Problems. *Int J Family Med* 2011;2011:490634. doi: 10.1155/2011/490634 [published Online First: 2012/02/09]
40. Rogers A, Kennedy A, Nelson E, et al. Uncovering the limits of patient-centeredness: implementing a self-management trial for chronic illness. *Qual Health Res* 2005;15(2):224-39.

For peer review only - http://bmjopen.bmj.com/site/about/guidelines.xhtml

UOB Confidential

41. Lamb J, Bower P, Rogers A, et al. Access to mental health in primary care: a qualitative meta-synthesis of evidence from the experience of people from 'hard to reach' groups. *Health (London)* 2012;16(1):76-104.
42. Kovandzic M, Chew-Graham C, Reeve J, et al. Access to primary mental health care for hard-to-reach groups: from 'silent suffering' to 'making it work'. *Soc Sci Med* 2011;72(5):763-72.
43. Drury CA, Louis M. Exploring the association between body weight, stigma of obesity, and health care avoidance. *J Am Acad Nurse Pract* 2002;14(12):554-61.
44. Olson CL, Schumaker HD, Yawn BP. Overweight women delay medical care. *Arch Fam Med* 1994;3(10):888-92.
45. Throsby K. Obesity surgery and the management of excess: exploring the body multiple. *Sociol Health Illn* 2012;34(1):1-15.

For peer review only

For peer review only - http://bmjopen.bmj.com/site/about/guidelines.xhtml

UOB Confidential

Table 1: Characteristics of participants

Participant	Gender	Age range (years)	Marital status	Employment status	Type of surgery	Time since surgery (years)
P01	Female	60-70	Married	Retired	RYGB	>5
P02	Female	50-60	Married	Unemployed	RYGB	<1
P03	Female	30-40	Married	Employed*	RYGB	1-2
P04	Female	60-70	Married	Retired	AGB	>5
P05	Male	40-50	Married	Employed	RYGB	<1
P06	Female	30-40	Married	Employed	Awaiting surgery	N/A
P07	Female	40-50	Married	Employed	RYGB	>5
P08	Male	60-70	Married	Employed	AGB	>5
P09	Female	40-50	Married	Unemployed	SG	1-2
P10	Male	30-40	Co-habiting	Self-employed	SG	2-5
P11	Female	40-50	Married	Employed	SG	<1
P12	Female	50-60	Married	Self-employed	SG	1-2
P13	Male	50-60	Widowed	Employed	RYGB	<1
P14	Female	40-50	Married	Employed	AGB & RYGB	>5
P15	Male	60-70	Married	Retired	RYGB	1-2
P16	Female	60-70	Married	Retired	Awaiting surgery	N/A
P17	Male	40-50	Married	Employed	AGB	2-5
P18	Male	50-60	Co-habiting	Employed	AGB	1-2
P19	Female	30-40	Separated	Employed	AGB	1-2

AGB=Adjustable gastric band, RYGB=Roux-en-Y gastric bypass, SG=Sleeve gastrectomy

***'Employed' status includes those employed both full-time and part-time**

For peer review only

For peer review only - http://bmjopen.bmj.com/site/about/guidelines.xhtml

UOB Confidential

Table 2: Participant quotes to support positive experiences of follow-up care

Positive aspects of care	**Quotes**
Routine monitoring of certain measures	“It was good having my bloods done so I could check what my levels were like, that was quite useful for me…routine monitoring was good.” (P07)
The availability of a key health professional; Ability to contact the team using a range of contact options	“If I couldn’t get hold of her (dietitian) straight away on the phone I’d send an email and it would either be answered the same day or the next day.” (P09)
Good communication between team members	“It’s quite a tight little team….you might not necessarily speak to the best person, but they will come together in their meeting and you’ll get the best outcome.” (P19)
Continuity of care	“You didn’t see twenty different people. It was ‘the team’…the same faces…I like that. I don’t want to see somebody who’s different don’t know you…” (P08)
Overall positive view of care	“The follow-up care I’ve had has just been 110%, if I’ve had a problem I would ring and…I would get an appointment…Someone has always been there for me…” (P01)

For peer review only

For peer review only - http://bmjopen.bmj.com/site/about/guidelines.xhtml

UOB Confidential

Document S1 (Study protocol)

The patient perspective of living with surgery for morbid obesity: Creating a patient 'core' outcome set, and investigating ways to improve follow-up care

Chief Investigator

Karen D Coulman, NIHR Doctoral Research Fellow, School of Social and Community Medicine, Unversity of Bristol

Supervisors

Dr Amanda Owen-Smith, School of Social and Community Medicine, Unversity of Bristol

Professor Jane M Blazeby, School of Social and Community Medicine, Unversity of Bristol

Funding

National Institute of Health Research (NIHR) Doctoral Research Fellowship award

For peer review only

For peer review only - http://bmjopen.bmj.com/site/about/guidelines.xhtml

Lay Summary

Surgery is increasingly being used as a treatment for obesity. Studies report many different outcomes, often measured in different ways, and little is known about what outcomes are important to patients. This study aims to explore the outcomes that are important to patients and to use this information to develop a short list of the most important outcomes to health professionals and patients (a core outcome set), which can be used to evaluate surgical treatments for obesity. In addition, this study will investigate patients' experiences of obesity surgery to provide recommendations for how post-operative support can be improved in the NHS. This will be achieved by interviewing obesity surgery patients to determine whether published outcomes reflect those outcomes patients themselves consider important, and to obtain views on living with surgery and ways to improve post-operative support. A list of patient-centred outcomes will be created from the literature and the interviews which will be added to a list of 'medical' outcomes of obesity surgery. This long list of outcomes will be reduced into a "core" set of outcomes, using a scientific process to reach agreement, which involves 2 rounds of questionnaires completed by health professionals and patients. The final core outcome set will be agreed in separate consensus meetings with a small group of health professionals and patients. This core outcome set will be useful to researchers in choosing outcomes for research trials that are important to both health professionals and patients. Use of the core outcome set would also improve the overall quality of reporting in trials. This research will also be useful for health professionals to ensure health services are designed based on patient priorities.

For peer review only

Background

Obesity surgery

The NHS Information Centre has released figures showing that just over one quarter of the adult population of England was obese in 2010, an increase from 13-16% of the population in 1993[1]. It is predicted that one third of adults in England will be obese by 2015 and more than 700 million adults will be obese worldwide by 2015[1;2]. Obesity is associated with an increased risk of type 2 diabetes, heart disease, cancer, reduced quality of life and premature death[1;3]. As such, obesity is a major public health concern. Various treatments for obesity exist including lifestyle modifications, pharmacotherapy, and more recently, surgical interventions. Obesity surgery operations have rapidly increased in the UK, and worldwide, with the most common operations being the adjustable gastric band and the Roux-en-Y gastric bypass[1;4]. In 2009, a Health Technology Assessment report (including a Cochrane systematic review) concluded that obesity surgery is more clinically effective than other treatments for obesity in terms of weight loss, improvement of co-morbidities such as type 2 diabetes and hypertension, and is also cost effective[5].

The patient perspective of outcomes of obesity surgery

Although clinical outcomes are important to measure, patient-reported outcome (PRO) measures, such as measures of health-related quality of life (HRQL), provide a means for capturing how a patient feels about their health or condition[6]. The Food and Drug Administration (FDA) has published guidance on the development of PRO measures to support claims of treatment benefit[6]. PRO measures should be developed based on rigorous qualitative research with patients to ensure content validity (authenticity to patients)[6;7]. Despite this, the development of PRO measures is variable and not always transparent[7].

The lead researcher has recently undertaken a systematic review of PROs in obesity surgery studies, which identified 68 different validated questionnaires used to assess PROs amongst the 86 identified studies [8]. Due to the heterogeneity of the items and scales within the questionnaires used amongst the various studies, a meta-analysis to determine the effect of obesity surgery on PROs was unable to be undertaken. The lead researcher then looked in further depth at the development of those questionnaires which were classified as obesity-specific (19 questionnaires). Although 14 (74%) reported to have involved patients in questionnaire development, only 3 (16%) specified that obesity surgery patients were involved. Only 6 (32%) specified that qualitative interviews/focus groups were used to obtain patient views and none specified that a qualitative analysis of resulting data was undertaken. Thus, it is not certain whether the majority of PRO questionnaires being used to assess obesity surgery are grounded in patient views.

Although well-developed PRO measures can provide valuable information about the patient's perspective of outcomes of treatment, primary qualitative studies can also yield rich information; however, these types of studies are rarely included in Cochrane reviews of quantitative evidence. The lead researcher is currently undertaking a review of qualitative research studies where the patient perspective of obesity surgery was sought. Although qualitative studies have focused on patient experiences of surgery, none have explicitly investigated patients' views of important outcomes of obesity surgery.

There is clearly more work needed to clarify the outcomes of obesity surgery from the patient perspective.

A 'core' outcome set for obesity surgery

As described above, heterogeneity of PROs in obesity surgery studies is an issue, which limits cross-study comparison and amalgamation of study results, which in turn limits the ability to make recommendations to clinicians and policymakers about the impact of obesity surgery on outcomes

For peer review only - http://bmjopen.bmj.com/site/about/guidelines.xhtml

important to patients. This issue is not unique to PROs, but also to clinical outcome measures with a Cochrane review of obesity surgery unable to undertake a meta-analysis due to a lack of consistency in outcomes reported[5].

Recent years have seen an increasing interest in the establishment of 'core outcome measures' to promote consistency in the reporting of clinical trials. Of particular interest is the development of the COMET (Core Outcome Measures in Effectiveness Trials) initiative through the MRC Hubs for Trials Methodology Research whose aim is to bring together researchers interested in the development and application of core outcome sets for specific conditions[9;10](1). Another notable initiative is OMERACT (Outcome Measures in Rheumatology Clinical Trials) in rheumatology[11]. Core outcome sets are not meant to be a restrictive list, but a minimum set of outcomes that should be reported on in every trial of a particular disease/condition. By standardising outcomes to be reported on in trials, results can be compared, contrasted and synthesised (e.g. meta-analysis) to inform health policy. No core outcome set for obesity surgery currently exists.

Recent research demonstrates that patients' perceptions of important outcomes may differ considerably from clinicians' perspectives, and therefore incorporating the patient perspective is crucial to contribute to and validate core outcome sets[12]. Creating a core outcome set for obesity surgery based on both health professional and patient views will help in the ordering of research and health service priorities to include the patient perspective, and ultimately improve long-term outcomes.

The patient perspective of follow-up care after obesity surgery

Follow-up care after obesity surgery varies greatly across centres. There is no consensus as to what optimum follow-up care is, although most clinicians recognise that good follow-up care is integral in achieving good outcomes from obesity surgery[13-15]. Understanding the outcomes of most importance to patients, and their experiences of follow-up care would provide invaluable information to design effective follow-up care programmes tailored to patients needs to maximise the benefits of surgery in the long-term. Qualitative research with obesity surgery patients in the UK context has focused on the experiences of patients, including difficulties they encounter after obesity surgery[16;17] but to our knowledge, no studies have specifically focused on patients' experiences of follow-up care and ways to improve follow-up care in the health services.

There is clearly more work needed to clarify optimum follow-up care from the patient perspective, and its influence on outcomes. As obesity and the number of obesity surgery operations increase, it is imperative that research in this area is of high quality, and takes into account the patient perspective.

Aims

The overall aims are to develop a core outcome set for obesity surgery including both health professional and patient perspectives and to investigate patients' experiences of living with the results of obesity surgery including ways to improve post-operative follow-up care.

Objectives

1. Semi-structured qualitative interviews with obesity surgery patients will be conducted to:
    a. Investigate outcomes of importance to obesity surgery patients that may not have been documented in the literature previously, and to add these to a comprehensive list of PROs of obesity surgery previously compiled from systematic literature reviews.
    b. Investigate patients' experiences of living with obesity surgery and ways to improve post-operative support.

For peer review only - http://bmjopen.bmj.com/site/about/guidelines.xhtml

2. The list created in objective 1a above will be added to a list of clinical outcomes which will be used within a Delphi survey of health professionals and patients which will ask them to prioritise the outcomes and produce a shorter list. The short list will be considered at separate meetings with health professionals and patients who will agree and finally ratify the content of the core outcome set.

Methods

Semi-structured qualitative interviews

To supplement the literature reviews previously undertaken by the lead researcher, patients' views of important outcomes of obesity surgery and experiences of existing follow up care will be investigated using semi-structured interviews within a qualitative paradigm.

Identification and selection of patients
Patients who are about to undergo and who have undergone a primary operation for obesity will be identified by health professionals from participating obesity surgery services at NHS healthcare Trusts using departmental databases and clinic lists. The main criteria for selection will be: The patient is undergoing obesity surgery within the next three months (pre-surgery group), or has undergone obesity surgery (post-surgery group). Including patients at both the pre- and post-operative stage will allow us to compare views and assess differences. Patients will initially be purposively sampled to obtain maximum variation for gender, age, ethnicity, starting BMI, type of operation, and time since operation, however further sampling will be guided by emerging findings from interviews.

Patient recruitment
Identified patients will be sent a letter from their local NHS obesity surgery team including an invitation letter and patient information sheet from the lead researcher, informing them of the research and inviting them to participate in the interviews. A reply slip and stamped addressed envelope will be included for patients to indicate whether or not they are interested in participating in the research to post back to the lead researcher. The number of patients recruited will depend on when theoretical saturation is reached (i.e. when the themes relevant to the research have been thoroughly investigated); however it is estimated that approximately 30 patients will be interviewed in total.

Data collection and analysis
Patients who agree to take part in the interviews will be able to choose the location of the interview, either in their own home, at one of the two participating hospitals, or the University of Bristol. Should any participants request to be interviewed at their GP surgery instead, permission will be sought by the chief investigator from the surgery's practice manager to conduct the interview at the surgery. Any travel costs will be reimbursed. Consent will be obtained face-to-face prior to the interview beginning. Should any participants request to be interviewed via telephone, participants will be mailed two copies of the consent form and asked to sign and return them to the chief investigator who will then sign them and return one copy to the participant prior to the interview. An interview topic guide will be applied flexibly to guide interviews. The following broad themes will be explored in interviews: 1) Motivations to undergo surgery; 2) Expected outcomes of surgery; 3) Actual outcomes of surgery (post-op group only); 4) Expectations of follow-up care; 5) Actual experiences of follow-up care (post-op group only). It is anticipated that interviews will last approximately 60 minutes, including 10 minutes dedicated to recording socio-demographic and relevant clinical data at the end of the interview. Interviews will be audio-recorded and transcribed in full. Separate consent will be sought to potentially re-contact them in the future for a follow-up interview.

For peer review only - http://bmjopen.bmj.com/site/about/guidelines.xhtml

Interviews will be analysed thematically using a grounded theory approach[18]. Data will be analysed using descriptive and explanatory coding to explore and inter-relate categories arising in the data. Data management will be facilitated using the program NVivo 9[19]. Descriptive accounts will be written up relating to each batch of interviews, and matrices will be drawn up to compare the occurrence of themes across interviews. Data analysis will run in parallel with data collection so that emerging themes can be followed up to enrich subsequent interviews. A small sample of the interview transcripts will be independently coded by both the chief investigator and her primary supervisor. The coding structures will then be discussed in a supervision meeting and revised as appropriate. The analysis will also be reviewed with the chief investigator's co-supervisor, and patient research partners.

Findings from the qualitative interviews will be combined with findings from a previous review of qualitative literature in this area, and written up for publication as soon as possible. Particular attention will be paid to ensuring that recommendations relating to the improvement of follow-up care are disseminated as soon as possible to clinicians and policymakers.

Important outcomes identified by patients in the semi-structured interviews will be added to the outcomes previously identified from systematic literature reviews undertaken by the lead researcher to create a comprehensive 'long' list of outcomes important to obesity surgery patients. The list will be reviewed by the lead researcher, her supervisors, and patient research partners to make sure it is comprehensive such that potentially important outcomes have not been omitted. This will form the basis of the next stage of the research (Delphi process).

Delphi process

This comprehensive list of outcomes will be refined into a 'core' outcome set using a Delphi process consisting of two questionnaire rounds where health professionals and patients individually rate each outcome for its value of being included in the core outcome set, followed by consensus meetings to discuss any potential areas of disagreement with regard to which outcomes to include and to ratify the final core outcome set.

Ethical issues arising

Informed consent

Written, informed consent will be obtained from all participants prior to the qualitative interviews. Confidentiality and anonymity will be assured at this stage and the purposes and possible uses of the research will be explained. It will be made clear that participants are free to drop out or delay participation in research at this stage. Separate consent will be sought to digitally-record interviews, and to potentially re-contact the participants in future for a follow-up interview.

Data protection

The principles of the Data Protection Act (1998) will be complied with and data obtained from interviewees will be anonymised using unique study codes. Documents to interpret the codes and personal data will be stored in separate encrypted files in separate locations on the University of Bristol server. All hard copy study documents will be stored in locked filing cabinets. Only data necessary to the purposes of the research will be obtained and stored.

For peer review only - http://bmjopen.bmj.com/site/about/guidelines.xhtml

Confidentiality

Confidentiality and anonymity outside of the interviews will be discussed and assured prior to beginning each interview. It will also be assured through the processes of data storage outlined above and the use of only fully anonymised quotations in all presentations of research findings.

Protection from distress

It is understood that living with morbid obesity and making the decision to undergo obesity surgery are personal and sensitive issues for patients. Therefore interviews will be undertaken sensitively and patients will only be encouraged to talk about aspects of their illness and care that they feel comfortable with. In the event that a participant becomes distressed during a research interview, the researcher will offer to take a short break from the interview and will remind participants that they do not have to discuss any topics they are uncomfortable with and are free to withdraw their consent to participate in the research at any time. If any patients express particular distress relating to their condition or treatment, a distress protocol will be followed (Appendix 1). The interviewer previously worked as a health professional in a NHS obesity surgery service and has experience of dealing with sensitive issues that may be brought up by this patient population. In addition, the interviewer will be supervised by a trained qualitative researcher who has experience in interviewing morbidly obese patients.

Integrity of research data

The MRC principles of Good Research Practice will be adopted with all primary data being retained so that there is an auditable trail from results back to data. Results will be fully and accurately reported at the end of the project.

Independence and impartiality

Every attempt will be made to maintain reflexivity at every stage of the research and to acknowledge the potential impact of the researcher upon the findings.

For peer review only

For peer review only - http://bmjopen.bmj.com/site/about/guidelines.xhtml

Appendix 1: Distress protocol - Interviews

All interviews will be prefaced with a statement about confidentiality and the duty of care. Participants will be told that interview is strictly confidential but should they disclose information to suggest that they are at significant risk of harm the researcher *may* have to discuss this with a clinical advisor.

In the event that a participant appears to be distressed during the interview (eg. becomes silent, cries) or discloses information to provoke concern about suicide risk, the following procedures will be followed:

- Participants will be offered the opportunity to pause for a break from the interview and will then be asked if they would like to resume.
- If necessary, the interview will be terminated and recording equipment stopped.
- At first, the interviewer will listen to the interviewee and offer support in situ. This will allow the researcher to assess whether further action is necessary.
- Should the interviewer remain concerned, they will reflect this to the interviewee and depending on the nature of the situation:
    1. Offer information about local help services
    2. Ask the interviewee if there is anyone they should contact, and if so attempt to make contact
    3. Offer to make initial contact with clinical services (primary or secondary) on behalf of the individual and with their consent
- In cases of particular concern the interviewer will
    1. If necessary, remain with the person until their distress has subsided or someone else is present
    2. Contact a local study clinician for advice/ assistance
    3. Provide a written report of the incident to AOS/JB (PhD supervisors), including information about the nature of the distress and the actions taken

Interviewees will be advised to contact their GP should they find subsequently that the interview provokes issues that they need to discuss.

For peer review only - http://bmjopen.bmj.com/site/about/guidelines.xhtml

Reference List

(1) The NHS Information Centre, Lifestyles Statistics. Statistics on obesity, physical activity and diet: England, 2012. The Health and Social Care Information Centre.

(2) World Health Organization. Fact sheet No. 311: Obesity and overweight. 2006.

(3) Must A, Spadano J, Coakley EH, Field AE, Colditz G, Dietz WH. The disease burden associated with overweight and obesity. *JAMA* 1999; 282(16):1523-1529.

(4) Buchwald H, Oien DM. Metabolic/bariatric surgery Worldwide 2008. *Obesity Surgery* 2009; 19(12):1605-1611.

(5) Picot J, Jones J, Colquitt JL, Gospodarevskaya E, Loveman E, Baxter L et al. The clinical effectiveness and cost-effectiveness of bariatric (weight loss) surgery for obesity: a systematic review and economic evaluation. *Health Technology Assessment* 2009; 13(41):1-+.

(6) U.S.Department of Health and Human Services FDA Center for Drug Evaluation and Research, U.S.Department of Health and Human Services FDA Center for Biologics Evaluation and Research, U.S.Department of Health and Human Services FDA Center for Devices and Radiological Health. Guidance for industry: patient-reported outcome measures: use in medical product development to support labeling claims: draft guidance. *Health & Quality of Life Outcomes* 2006; 4:79.

(7) Lasch KE, Marquis P, Vigneux M, Abetz L, Arnould B, Bayliss M et al. PRO development: rigorous qualitative research as the crucial foundation. *Quality of Life Research* 2010; 19(8):1087-1096.

(8) Coulman KD, Abdelrahman T, Owen-Smith A, Andrews RC, Welbourn CR, Blazeby JM. Measuring patient reported outcomes in bariatric surgery: standards of reporting and synthesis with clinical data. *Br J Surg* 2012; 99(Suppl. 2):1.

(9) University of Liverpool. COMET Initiative: http://www.comet-initiative.org/. 2012.

(10) Medical Research Council. MRC Hubs for Trial Methodology Research: http://www.methodologyhubs.mrc.ac.uk/. 2012.

(11) Tugwell P, Boers M, Brooks P, Simon L, Strand V, Idzerda L. OMERACT: An international initiative to improve outcome measurement in rheumatology. *Trials* 2007; 8.

(12) Hewlett SA. Patients and clinicians have different perspectives on outcomes in arthritis. *Journal of Rheumatology* 2003; 30(4):877-879.

(13) Myers VH, Adams CE, Barbera BL, Brantley PJ. Medical and Psychosocial Outcomes of Laparoscopic Roux-en-Y Gastric Bypass: Cross-sectional Findings at 4-Year Follow-up. *Obesity Surgery* 2012; 22(2):230-239.

(14) American Society for Metabolic & Bariatric Surgery. ASMBS Position Statement on Global Bariatric Healthcare. 2011.

For peer review only - http://bmjopen.bmj.com/site/about/guidelines.xhtml

(15) Heber D, Greenway FL, Kaplan LM, Livingston E, Salvador J, Still C. Endocrine and Nutritional Management of the Post-Bariatric Surgery Patient: An Endocrine Society Clinical Practice Guideline. *Journal of Clinical Endocrinology & Metabolism* 2010; 95(11):4823-4843.

(16) Ogden J, Clementi C, Aylwin S. The impact of obesity surgery and the paradox of control: A qualitative study. *Psychology & Health* 2006; 21(2):273-293.

(17) Ogden J, Clementi C, Aylwin S, Patel A. Exploring the impact of obesity surgery on patients' health status: a quantitative and qualitative study. *Obesity Surgery* 2005; 15(2):266-272.

(18) Glaser B, Strauss A. The discovery of grounded theory: Strategies for qualitative research. Chicago: Aldine; 1967.

(19) NVivo 9, QSR International; 2010.

For peer review only

For peer review only - http://bmjopen.bmj.com/site/about/guidelines.xhtml

Document S2: Topic guides

Pre-operative patient interviews

NB This guide is necessarily provisional, as its application will depend on the experience of individual participants.

**Introduction**<br>❖ *Researcher explains research, asks if any queries on PIS, and takes consent.*
**Motivations to undergo surgery**<br>- To start off, I was just wondering if you could tell me a bit about what factors led to your decision to go for surgery?
**Expected outcomes of surgery**<br>- How are you feeling about going forward for surgery now?<br>*Probe: Which operation are you hoping to have?*<br>*Probe: Is there anything you're worried about? [Prompt on risks/side-effects of surgery]*<br>*Probe: What do you think will change for the better after you have had surgery?*<br>*Probe: Is there anything you are hoping to prevent by having surgery?*<br>- To you, what are the most important results of surgery?<br>*If they say weight loss, Probe: What does weight loss mean for you?*<br>*Probe on any aspects they don't elaborate on eg. What does that mean for you?*<br>*Probe: Would you say these are the same things that other people undergoing weight loss surgery think are important?*<br>- Are there any things about the surgery or life afterwards that you would have liked the healthcare team to give you more information about?<br>*Probe: Do you think other people who are undergoing surgery would say the same things?*
**Experiences of care so far and expectations of follow-up care**<br>- What happened when you decided to go for surgery?<br>*Probe: Where did you first hear about surgery?*<br>- *Probe: When did you make the decision to put yourself forward for surgery?*<br>*Probe: Who did you talk to first?*<br>*Probe: What happened next? [Prompt on healthcare professionals they have seen [GP practice, local weight management team, surgical team]*<br>*Probe: Would you say this is the same or different to the process that others have gone through?*<br>- How do you feel about the care/support you have received from the healthcare team around weight loss surgery up until this point? *[Prompt on the surgical team, the local weight management team, GP practice]*<br>*Probe: Do you think others have had similar experiences?*<br>- Do you have any ideas about the care you will receive after surgery? *[Prompt on short-term inpatient care and long-term care]*<br>- Could you say at this stage what kind of care you're hoping for after surgery?<br>*Probe: Who would be the best person/people to do that?*<br>*Probe: What would be the best setting for this? [Prompt on hospital/ another setting]*<br>*Probe: Would this be the same as what others would say?*<br>- What about support groups – have you been involved with any of these?<br>*If yes Probe: Do you tend to go to meetings or do you use online forums?*<br>*Probe: How do you find these? [Prompt on positive and negative aspects]*<br>*If no Probe: What's stopped you from getting involved?[ie. prompt on if unaware of them, none convenient, don't find helpful]*
**Clarifications, etc.**<br>❖ *Researcher asks participant to provide more information where points of interest were not previously probed.*
**Wind-down**<br>❖ *Researcher checks topic guide for omissions and prompts informant back towards areas of discussion where they seemed most comfortable.*
**Case report form**<br>❖ *Researcher records participant details using the case report form.*
**Closing**<br>❖ *Researcher asks if patient would be interested in being contacted about a possible follow-up interview – to be recorded on consent form*

Version 1.2 (18/3/2013)

For peer review only - http://bmjopen.bmj.com/site/about/guidelines.xhtml

- ❖ *Researcher thanks interviewee for participating and asks if they would like a summary of the study results once complete*

For peer review only

For peer review only - http://bmjopen.bmj.com/site/about/guidelines.xhtml

Post-operative patient interviews

NB This guide is necessarily provisional, as its application will depend on the experience of individual participants.

Introduction

❖ *Researcher explains research, asks if any queries on PIS, and takes consent.*

Motivations to undergo surgery

- To start off, I was just wondering if you could tell me a bit about what factors led to your decision to go for surgery?

Expected outcomes of surgery

- If you can think back to a few weeks before you had the surgery, can you remember how you were feeling about having surgery?
  Probe: Was there anything you were worried about? [Prompt on risks/side-effects of surgery]
  Probe: Was there anything you hoped would change for the better by having the surgery?
  Probe: Was there anything you were hoping to prevent by having surgery?

Actual outcomes of surgery

- How do you feel about having had surgery now?
  Probe: Which operation did you have?
  Probe: How long ago was it now?
  Probe: What's happened since? [Prompt on the things they said they were worried about/hoped would change/hoped they would prevent by having surgery]
  Probe: Was there anything that happened that you didn't expect? [Prompt on during surgery and after surgery]
  Probe: Are you glad you had the surgery?
  Probe: Do you think having the operation has changed your relationships with people who are important to you?
- To you, what are the most important results of surgery?
  If they say weight loss, Probe: What does weight loss mean for you?
  Probe: Are these things different to what you would have said before having surgery?
  Probe: What results of surgery might be most important to other people undergoing the surgery?
  If yes, Probe: How are they different?
- Are there any things that you didn't know beforehand that you wished you had?
- What do you think will happen in the future?

Expectations of follow-up care

- If you can think back to a few weeks before you had the surgery, can you remember if you had any expectations or ideas about the care you would receive after surgery? *[Prompt on short-term inpatient care and long-term care]*

Actual experiences of follow-up care

- Since you've had your surgery, have you been back to the hospital? *[Prompt on the surgical and weight management teams,]*
  Probe: What about your GP practice, have you seen anyone there since your surgery?
  Probe: Do you think there is a role for more follow-up care from primary care?
- What do you think will happen next?
- How do you feel about the NHS care you have received since your surgery?
  Probe: Tell me about the things you found most helpful
  Probe: Tell me about the things you found least helpful
  Probe: Is there anything you would have preferred to have been done differently?/Is there anything you think the NHS could have done to better support you since you've had your surgery?
- Could you say what you thought the most important things of good care after weight loss surgery were?
  Probe: Who would be the best person/people to do that?
  Probe: What would be the best setting for this? [Prompt on hospital/ another setting]
  Probe: Do you think these are the same things that others who've had surgery would say?
- What about support groups – have you been involved with any of these?
  If yes Probe: Do you tend to go to meetings or do you use online forums?
  Probe: How do you find these? [Prompt on positive and negative aspects]
  If no Probe: What's stopped you from getting involved?[ie. prompt on if unaware of them, none convenient, don't find helpful]
  If used to be involved but now no longer involved Probe: What's led to this?

Clarifications, etc.

Version 1.2 (18/3/2013)

❖ *Researcher asks participant to provide more information where points of interest were not previously probed.*
**Wind-down**<br>❖ *Researcher checks topic guide for omissions and prompts informant back towards areas of discussion where they seemed most comfortable.*
**Case report form**<br>❖ *Researcher records relevant socio-demographic and clinical details using the case report form.*
**Closing**<br>❖ *Researcher asks if patient would be interested in being contacted about a possible follow-up interview – to be recorded on consent form*<br>❖ *Researcher thanks interviewee for participating and asks if they would like a summary of the study results once complete*

For peer review only

For peer review only - http://bmjopen.bmj.com/site/about/guidelines.xhtml

Document S3: Case Report Form - Patient views about weight loss surgery

Participant identification number:<br>Date of birth: __/__/____<br>Initials:<br>Today's date: __/__/____	*If patient has consented to GP being informed about participation in study*:<br>GP name:<br>GP surgery and address:

1.	What is the gender of the participant? 1=Male, 2=Female	☐
2.	What is the patient's ethnicity? 1=White British, 2=White other, 3=Black, 4=Asian, 5=Mixed, 6=Chinese, 7=Other	☐
3.	Who does the participant live with? 1=alone, 2=with family, 3=with other adults	☐
4.	What is the participant's marital status? 1=single, 2=married, 3=separated, 4=divorced, 5=widowed	☐
5.	What is the highest level of education completed? 1=less than compulsory school education, 2=compulsory school education, 3=post-compulsory school education below university level, eg. advanced technical school/advanced vocational, 4=university level	☐
6.	What is the employment status of the participant? 1=employed full-time (could be on sick leave), 2=employed part-time, 3=homemaker, 4=student, 5=unemployed, 6=retired, 7=self-employed, 8=other (specify)..	☐
7.	What is the current or last occupation of the participant?...	
8a.	What is the patient's surgical status? 1=Awaiting surgery, 2=Undergone surgery (go to 8e.)	☐
b.	If the participant is awaiting surgery, what operation are they hoping to undergo? 1=LAGB, 2=RYGB, 3=SG, 4=Other (specify)..	☐
c.	If the participant is awaiting surgery, do they have a date? 1=Yes, 2=No	☐
d.	If yes, what is the date?	__/__/____
e.	If the participant has undergone surgery, what operation have they undergone? 1=LAGB, 2=RYGB, 3=SG, 4=Other (specify)..	☐
f.	If the participant has undergone surgery, what date did it occur?	__/__/____
9a.	What was/is the participant's weight prior to surgery (self-reported)? ☐ Stones ☐ lbs ☐ Kg	
b.	If the participant has undergone surgery, what is their weight now (self-reported)? ☐ Stones ☐ lbs ☐ Kg	
10.	What is the participant's height (self-reported)? ☐ feet ☐ inches ☐ cm	

For peer review only - http://bmjopen.bmj.com/site/about/guidelines.xhtml

11.	Does the participant have any co-morbidities? List as many as needed. 1=diabetes, 2=hypertension, 3=hyperlipidaemia, 4=cardiac disease (excluding 2 and 3), 5=sleep apnoea, 6=asthma, 7=joint problems (eg. arthritis), 8=urinary incontinence, 9=infertility, 10=other, 11=None. If other, please specify..	☐ ☐ ☐ ☐

For peer review only

For peer review only - http://bmjopen.bmj.com/site/about/guidelines.xhtml

For peer review only

Document S4: Final coding structure used in NVivo

Name of code	Code related to
General NHS comments not related to surgery	Aspects of care
Guidelines from health professionals	Aspects of care
Follow-up care	Aspects of care
Factors leading to decision to have surgery	Life pre-surgery
Past struggles with weight	Life pre-surgery
Pre-op feelings about surgery	Life pre-surgery
Expectations of surgery	Life pre-surgery
Pre-surgery preparation	Life pre-surgery
Liver shrinking pre-op diet	Life pre-surgery
Other people's perceptions of obesity	Life pre-surgery
NHS funding of surgery	Life pre-surgery
Criteria to fulfill to obtain surgery	Life pre-surgery
Background	Life pre-surgery
Choice of operation	Life pre-surgery
Social impact of obesity	Life pre-surgery
Treatment from health professionals around obesity	Life pre-surgery
Other people's experiences of surgery	Life post-surgery
Future expectations and hopes	Life post-surgery
Peri-operative experience	Life post-surgery
Factors that help maintain motivation post-surgery	Life post-surgery
Support groups	Life post-surgery
Outcome - Activity, mobility	Life post-surgery
Outcome - Body image	Life post-surgery
Outcome - Comorbidity	Life post-surgery
Outcome - Eating	Life post-surgery
6 week post-op diet	Life post-surgery
Outcome - GI symptoms	Life post-surgery
Bowel movements	Life post-surgery
Wind or gas	Life post-surgery
Dumping or sickness	Life post-surgery
Outcome - Hunger	Life post-surgery
Outcome - Psychological	Life post-surgery
Outcome - Social impact of surgery	Life post-surgery
Family reactions to surgery	Life post-surgery
Outcome - Weight	Life post-surgery
In-hospital side-effects of surgery	Life post-surgery
Post-discharge side-effects of surgery	Life post-surgery
Most important outcomes of surgery	Life post-surgery
Outcome - Normality	Life post-surgery
Outcome - Clothing	Life post-surgery
Outcome - increased work options	Life post-surgery

For peer review only

COREQ checklist

No	Item	Guide questions/description	Location in text
**Domain 1: Research team and reflexivity**			
Personal characteristics			
1.	Interviewer/facilitator	Which author/s conducted the interview or focus group?	Page 6, line 9
2.	Credentials	What were the researcher's credentials? E.g. PhD, MD	Page 6, line 11
3.	Occupation	What was their occupation at the time of the study?	Page 6, line 11
4.	Gender	Was the researcher male or female?	Page 6, line 10
5.	Experience and training	What experience or training did the researcher have?	Page 6, line 11
Relationship with participants			
6.	Relationship established	Was a relationship established prior to study commencement?	Page 6, line 14
7.	Participant knowledge of the interviewer	What did the participants know about the researcher? e.g. *personal goals, reasons for doing the research*	Page 6, line 15
8.	Interviewer characteristics	What characteristics were reported about the interviewer/facilitator? e.g. *Bias, assumptions, reasons and interests in the research topic*	Page 6, lines 10-17
**Domain 2: Study design**			
Theoretical framework			
9.	Methodological orientation and Theory	What methodological orientation was stated to underpin the study? e.g. *grounded theory, discourse analysis, ethnography, phenomenology, content analysis*	Page 6, line 20

For peer review only

Participant selection			
10.	Sampling	How were participants selected? e.g. purposive, convenience, consecutive, snowball	Page 5, line 24
11.	Method of approach	How were participants approached? e.g. face-to-face, telephone, mail, email	Page 5, line 21
12.	Sample size	How many participants were in the study?	Page 7, line 10
13.	Non-participation	How many people refused to participate or dropped out? Reasons?	Page 7, line 10
Setting			
14.	Setting of data collection	Where was the data collected? e.g. home, clinic, workplace	Page 6, line 6
15.	Presence of non-participants	Was anyone else present besides the participants and researchers?	Page 7, line 11
16.	Description of sample	What are the important characteristics of the sample? e.g. demographic data, date	Page 7 lines 10-17, and Table 1
Data collection			
17.	Interview guide	Were questions, prompts, guides provided by the authors? Was it pilot tested?	Page 6, lines 7-8<br>Page 7, line 6
18.	Repeat interviews	Were repeat interviews carried out? If yes, how many?	Page 6, line 13
19.	Audio/visual recording	Did the research use audio or visual recording to collect the data?	Page 6, line 10
20.	Field notes	Were field notes made during and/or after the interview or focus group?	Page 6, line 17
21.	Duration	What was the duration of the interviews or focus group?	Page 7, line12

For peer review only

22.	Data saturation	Was data saturation discussed?	Page 6, lines 1-2
23.	Transcripts returned	Were transcripts returned to participants for comment and/or correction?	Page 6 line 19.
**Domain 3: Analysis and findings**			
Data analysis			
24.	Number of data coders	How many data coders coded the data?	Page 6, line 21
25.	Description of the coding tree	Did authors provide a description of the coding tree?	Page 6, line 23 and supplementary document S4
26.	Derivation of themes	Were themes identified in advance or derived from the data?	Page 6, lines 20-21
27.	Software	What software, if applicable, was used to manage the data?	Page 6, line 24
28.	Participant checking	Did participants provide feedback on the finding?	Page 7, lines 5-8
Reporting			
29.	Quotations presented	Were participant quotations presented to illustrate the themes/findings? Was each quotation identified? *eg. participant number*	Pages 7-13
30.	Data and findings consistent	Was there consistency between the data presented and the findings?	Pages 7-13
31.	Clarity of major themes	Were major themes clearly presented in the findings?	Pages 7-13
32.	Clarity of minor themes	Is there description of diverse cases or discussion of minor themes?	Pages 7-13

For peer review only

BMJ Open

Patients' experiences of life after bariatric surgery and follow-up care: A qualitative study.

Journal:	*BMJ Open*
Manuscript ID	bmjopen-2019-035013.R1
Article Type:	Original research
Date Submitted by the Author:	13-Dec-2019
Complete List of Authors:	Coulman , Karen ; University of Bristol, Population Health Sciences, Bristol Medical School<br>MacKichan, Fiona; University of Bristol, Population Health Sciences, Bristol Medical School<br>Blazeby, Jane; University of Bristol, Population Health Sciences, Bristol Medical School; University Hospitals Bristol NHS Foundation Trust, Division of Surgery, Head and Neck<br>Donovan, Jenny; University of Bristol, Population Health Sciences, Bristol Medical School; University Hospitals Bristol NHS Foundation Trust, NIHR CLAHRC West<br>Owen-Smith, Amanda; University of Bristol, Population Health Sciences, Bristol Medical School
**Primary Subject Heading**:	Qualitative research
Secondary Subject Heading:	Health services research, Qualitative research, Surgery
Keywords:	Organisation of health services < HEALTH SERVICES ADMINISTRATION & MANAGEMENT, Quality in health care < HEALTH SERVICES ADMINISTRATION & MANAGEMENT, QUALITATIVE RESEARCH, Adult surgery < SURGERY

SCHOLARONE™
Manuscripts

I, the Submitting Author has the right to grant and does grant on behalf of all authors of the Work (as defined in the below author licence), an exclusive licence and/or a non-exclusive licence for contributions from authors who are: i) UK Crown employees; ii) where BMJ has agreed a CC-BY licence shall apply, and/or iii) in accordance with the terms applicable for US Federal Government officers or employees acting as part of their official duties; on a worldwide, perpetual, irrevocable, royalty-free basis to BMJ Publishing Group Ltd ("BMJ") its licensees and where the relevant Journal is co-owned by BMJ to the co-owners of the Journal, to publish the Work in this journal and any other BMJ products and to exploit all rights, as set out in our licence.

The Submitting Author accepts and understands that any supply made under these terms is made by BMJ to the Submitting Author unless you are acting as an employee on behalf of your employer or a postgraduate student of an affiliated institution which is paying any applicable article publishing charge ("APC") for Open Access articles. Where the Submitting Author wishes to make the Work available on an Open Access basis (and intends to pay the relevant APC), the terms of reuse of such Open Access shall be governed by a Creative Commons licence – details of these licences and which Creative Commons licence will apply to this Work are set out in our licence referred to above.

Other than as permitted in any relevant BMJ Author's Self Archiving Policies, I confirm this Work has not been accepted for publication elsewhere, is not being considered for publication elsewhere and does not duplicate material already published. I confirm all authors consent to publication of this Work and authorise the granting of this licence.

For peer review only

Title: Patients' experiences of life after bariatric surgery and follow-up care: A qualitative study.

Authors: Karen D Coulman (karen.coulman@bristol.ac.uk)[1], Fiona MacKichan (fiona.mackichan@bristol.ac.uk)[1], Jane M Blazeby (j.m.blazeby@bristol.ac.uk)[1,2], Jenny L Donovan (jenny.donovan@bristol.ac.uk)[1,3], Amanda Owen-Smith (a.owen-smith@bristol.ac.uk)[1]

Affiliations: [1]Population Health Sciences, Bristol Medical School, University of Bristol, Bristol, UK

[2]Division of Surgery, Head and Neck, University Hospitals Bristol NHS Foundation Trust, Bristol, UK

[3]NIHR CLAHRC West, University Hospitals Bristol NHS Foundation Trust, Bristol, UK

Keywords: Adult surgery, Qualitative Research, Organisation of health services, Quality in health care

Running Title: Bariatric surgery and follow-up care

Corresponding author: Dr Karen Coulman, Population Health Sciences, Bristol Medical School, University of Bristol, 1-5 Whiteladies Road, Bristol, BS8 1NU, UK. Email: karen.coulman@bristol.ac.uk, Telephone: +44/0 117 42 83132.

Word count (main body of text excluding abstract, article summary, acknowledgements, references and tables): 4917

Funding: Karen D Coulman was funded by a National Institute for Health Research (NIHR) Doctoral Research Fellowship for this research project. This work was also supported by the Medical Research Council (MRC) ConDuCT-II Hub (Collaboration and innovation for Difficult and Complex randomised controlled Trials In Invasive procedures - MR/K025643/1). This publication presents independent research funded by the National Institute for Health Research (NIHR) and the MRC. The views expressed are those of the authors and not necessarily those of the National Health Service, the NIHR or the Department of Health and Social Care, or the MRC. JMB is an NIHR Senior Investigator.

Competing interests: None declared.

License statement: I, the Submitting Author has the right to grant and does grant on behalf of all authors of the Work (as defined in the below author licence), an exclusive licence and/or a non-exclusive licence for contributions from authors who are: i) UK Crown employees; ii) where BMJ has agreed a CC-BY licence shall apply, and/or iii) in accordance with the terms applicable for US Federal Government officers or employees acting as part of their official duties; on a worldwide, perpetual, irrevocable, royalty-free basis to BMJ Publishing Group Ltd ("BMJ") its licensees and where the relevant Journal is co-owned by BMJ to the co-owners of the Journal, to publish the Work in BMJ Open and any other BMJ products and to exploit all rights, as set out in our licence.

The Submitting Author accepts and understands that any supply made under these terms is made by BMJ to the Submitting Author unless you are acting as an employee on behalf of your employer or a postgraduate student of an affiliated institution which is paying any applicable article publishing charge ("APC") for Open Access articles. Where the Submitting Author wishes to make the Work available on an Open Access basis (and intends to pay the relevant APC), the terms of reuse of such Open Access shall be governed by a Creative Commons licence – details of these licences and which Creative Commons licence will apply to this Work are set out in our licence referred to above.

For peer review

FUNDED BY NIHR | National Institute for Health Research

For peer review only - http://bmjopen.bmj.com/site/about/guidelines.xhtml

UOB Confidential

Abstract

Objectives: Bariatric surgery is the most clinically effective treatment for people with severe and complex obesity, however, the psychosocial outcomes are less clear. Follow-up care after bariatric surgery is known to be important, but limited guidance exists on what this should entail, particularly related to psychological and social well-being. Patients' perspectives are valuable to inform the design of follow-up care. This study investigated patients' experiences of life after bariatric surgery including important aspects of follow-up care, in the long-term.

Design: A qualitative study using semi-structured individual interviews. A constant comparative approach was used to code data and identify themes and overarching concepts.

Setting: Bariatric surgery units of two publicly funded hospitals in the South of England.

Participants: Seventeen adults (10 women) that underwent a primary operation for obesity (mean time since surgery 3.11 years, range 4 months-9 years), including Roux-en-Y gastric bypass, adjustable gastric band and sleeve gastrectomy, agreed to participate in the interviews.

Results: Experiences of adapting to life following surgery were characterised by the concepts of 'normality' and 'ambivalence', while experiences of 'abandonment' and 'isolation' dominated participants' experiences of follow-up care. Patients highlighted the need for more flexible, longer-term follow-up care that addresses social and psychological difficulties post-surgery and integrates peer support.

Conclusions: This research highlights unmet patient need for more accessible and holistic follow-up care that addresses the long-term multi-dimensional impact of bariatric surgery. Future research should investigate effective and acceptable follow-up care packages for patients undergoing bariatric surgery.

FUNDED BY NIHR | National Institute for Health Research

For peer review only - http://bmjopen.bmj.com/site/about/guidelines.xhtml

UOB Confidential

Article Summary - strengths and limitations of this study

- Patients who had undergone all three main types of bariatric procedures across two UK centres were included in the research.
- A detailed qualitative approach was used, allowing participants to relate their own experiences in terms that were relevant for them.
- A rigorous approach to analysis was undertaken, including independent coding of initial transcripts by three researchers, and agreement of emergent themes throughout analysis with at least one other qualitative researcher.
- It is not known whether similar themes would be found with participants in other centres.
- Findings relating to follow-up care may be less generalisable to healthcare systems with different service pathways and funding structures.

For peer review only - http://bmjopen.bmj.com/site/about/guidelines.xhtml

FUNDED BY NIHR National Institute for Health Research

UOB Confidential

For peer review only

Introduction

Over 650 million or 13% of adults worldwide suffer from obesity (body mass index (BMI) ≥30 kg/m²), representing a tripling of figures since 1975.[1] Obesity is associated with an increased risk of type 2 diabetes, cardiovascular disease, certain cancers, and premature death.[2 3] Within this population, people with severe and complex obesity (BMI ≥40 kg/m2, or 35-40 kg/m2 with another significant health problem that could be improved by weight loss) suffer the greatest health burdens and are at the highest risk of premature death.[4 5] In addition to the physical and metabolic health burdens, people with severe and complex obesity are more likely to suffer with psychological disorders such as depression, anxiety and disordered eating, and reduced health-related quality of life (HRQL).[6 7] These individuals also suffer from social stigma and discrimination related to their weight [6 8] which is in turn associated with adverse physical and psychological outcomes.[8 9] Thus, any interventions to treat severe and complex obesity should consider the impact on these psychosocial outcomes in addition to traditional clinical and metabolic outcomes.[10 11]

Bariatric surgery, combined with behaviour change and dietary management, is the most clinically effective treatment for people with severe and complex obesity, in terms of weight loss and the improvement of co-morbidities such as type 2 diabetes.[5 12 13] The three main types of bariatric operations performed in the UK include the Roux-en-Y gastric bypass (RYGB, 53.9% in 2011-13), the sleeve gastrectomy (SG, 21.4%), and the adjustable gastric band (AGB, 21.4%).[14] More recent international data indicate that the SG (46.0%) and RYGB (38.2%) are the most common bariatric operations worldwide with AGB decreasing in recent years (5.0%), and the one-anastomosis gastric bypass now gaining popularity.[15] Each of these procedures works slightly differently; mechanisms include restriction in the amount of food able to be consumed, reduction in hunger, improvement in satiety, shift in food preferences, as well as altered gut hormones, bile acids, and vagal signalling.[16] Whilst there are lots of non-randomised studies in this field, there are very few well designed and

FUNDED BY NIHR | National Institute for Health Research

For peer review only - http://bmjopen.bmj.com/site/about/guidelines.xhtml

UOB Confidential

For peer review only

conducted randomised controlled trials with long-term follow-up. This means that true comparative assessments of RYGB, SG and AGB are absent from the literature. A current UK study has recently completed recruitment (n=1351), with the primary end point at three years. This will be the first pragmatic large-scale study examining all three procedures.[17]

Studies which have examined HRQL after each procedure are often poorly conducted with few including baseline data and comprehensive assessments of HRQL. Some show certain aspects of HRQL to improve but not others.[11 12 18] Previous qualitative research has highlighted the complex and changeable nature of the psychosocial impact of bariatric surgery, helping to shed light on some of these inconsistencies in the HRQL literature, and emphasising the importance of long-term post-operative support in helping patients manage these changes.[10 19] Previous research has also reported attendance at follow-up visits to be associated with better weight loss outcomes after bariatric surgery.[20] Follow-up care is thus important to optimise clinical and psychosocial outcomes of bariatric surgery. However, bariatric surgery follow-up care has been reported to vary greatly across the UK,[21] and current UK and US bariatric surgery guidelines focus on surgical and metabolic outcomes, with limited guidance on how to support psychological, social and lifestyle changes that affect patients' HRQL.[5 22] Nevertheless, previous work has highlighted the importance of these multi-faceted aspects of HRQL to patients that have undergone bariatric surgery and recommendations are needed on how best to support patients after surgery to optimise these outcomes.[23]

In seeking to evaluate and provide recommendations on bariatric surgery follow-up care, the patient's perspective can provide valuable information.[24] Qualitative research is useful to explore patients' perspectives as it seeks to gain the insider's view on how people view, experience, and make sense of their social world.[25-27] The primary focus of most previous qualitative research in bariatric surgery has been on patient experiences of outcomes of surgery rather than experiences of follow-up care.[10 19] Studies that have reported on aspects of care have identified patient need for longer follow-up after bariatric surgery, better access to psychological support, and the ability to

FUNDED BY NIHR | National Institute for Health Research

For peer review only - http://bmjopen.bmj.com/site/about/guidelines.xhtml

UOB Confidential

communicate with health professionals between routine appointments.[19 28-36] However, most of these studies were single-centre[29-32 34 36] or reported findings from select groups, such as patients that had undergone one type of bariatric procedure only (e.g. adjustable gastric band)[29 30 32-35] or had experienced negative outcomes such as weight re-gain or substance abuse issues.[28 29 32 34] A recent systematic review by Parretti *et al* identified few studies focusing on patients' experiences of follow-up care after bariatric surgery in the longer-term, and recommended that primary studies in this area were needed.[19] The objectives of this study were to: 1) Investigate experiences of life after bariatric surgery including follow-up care in the long-term across people that had undergone all three main types of UK bariatric procedures, and 2) Use these findings to provide recommendations for follow-up care.

Methods

Patients who had undergone a primary operation for obesity at two publicly funded bariatric surgery centres in the South of England were eligible to participate in the research. Patients were identified by health professionals at each hospital using databases and clinic lists and sent information about the research. Interested patients contacted the researcher directly (KDC). For initial interviews, patients were sampled purposively, aiming for maximum variation in gender, age, starting body mass index (BMI), type of operation, and time since operation. Emerging findings from analysis of initial interviews guided sampling for remaining interviews.[37] Sampling continued until themes were well-established with few or no new insights gained from additional data collection.[26 37] Ethical approval for the study was obtained from Northwest - Preston Research Ethics Committee (Ref 12/NW/0844). This study was undertaken as part of a wider study to develop a core outcome set for bariatric surgery (see **document S1** for protocol).[23]

Interviews were chosen as the method of data collection for this study due to the sensitive and complex nature of living with bariatric surgery, and to allow individual participants' experiences to

FUNDED BY NIHR | National Institute for Health Research

For peer review only - http://bmjopen.bmj.com/site/about/guidelines.xhtml

UOB Confidential

be explored in detail. Interviews were semi-structured to provide some consistency in topics discussed between interviews, while allowing flexibility to adapt each interview to the participant. Thirteen participants were interviewed in their homes, four in a private research room at one of the two participating hospitals, one in a private room at the University, and one over the telephone at their request. Interviews lasted between 44 and 110 minutes.

Written informed consent was taken and interviews conducted according to an outline topic guide, which evolved iteratively as the research progressed (see **document S2** for final version). Findings reported in this paper mainly relate to the sections of the topic guide 'Actual outcomes of surgery' and 'Actual experiences of follow-up care'. Relevant demographic and clinical information were also collected (**document S3**). All interviews were conducted and audio-recorded between February 2013 and November 2014, by a female researcher (KDC) who was a PhD student and registered dietitian. KDC underwent training in qualitative research methods and was supervised by two experienced qualitative researchers (AOS, FM). An initial telephone conversation was held with each participant to discuss the study and arrange the interview. Participants were otherwise not previously known to the researcher prior to interview. The researcher introduced herself as a PhD student to participants. She did not reveal her professional background as a registered dietitian unprompted but did not seek to hide it if participants asked. Field notes, which provided important contextual information to aid data analysis, were made as soon as possible after each interview.[38]

Recorded interviews were transcribed verbatim, and transcriptions checked for accuracy by KDC. Thematic analysis was undertaken, using techniques of constant comparison to code data and identify emerging themes.[37 39] As the aim of the study was to broadly investigate patients' experiences of surgery, including outcomes and aspects of care, this inductive approach to analysis was chosen to ensure that themes developed were strongly linked to the data. Coding was completed for all transcripts by KDC, with a sample of transcripts independently coded by two other experienced qualitative researchers (AOS and JLD) (see **document S4** for final coding framework).

FUNDED BY NIHR | National Institute for Health Research

For peer review only - http://bmjopen.bmj.com/site/about/guidelines.xhtml

UOB Confidential

Differences in interpretation were resolved through discussion. Initial codes were built into coding structures and themes were identified. Coding and data management were facilitated using NVivo 10 software.[40] Detailed descriptive accounts were written by KDC for each small batch of interviews, which described data relating to each theme and its constituent codes. It was at this stage that relationships between themes were identified, leading to the development of higher-order categories which encompassed inter-related themes. The coding and descriptive account were completed for each batch of interviews prior to recruiting additional patients so that emerging themes could be followed up to enrich subsequent interviews. Finally, large matrices were created to compare themes and categories across all participants and summary descriptive accounts were written wherein the concepts overarching all themes and categories crystallized.[39]. AOS, FM, JLD and JMB reviewed all descriptive accounts and made suggestions about further links between themes, categories and concepts.

Patient and Public Involvement

The idea for this research was based on the lead author's experience of working with patients over several years in a bariatric surgery service, as well as discussion with a representative from a relevant patient charity. This patient representative reviewed and provided feedback on the research proposal submitted for funding. After the study received funding, two patients who had undergone NHS-funded bariatric surgery were recruited as patient research partners and reviewed and provided feedback on the interview topic guide, and all written patient information (including study recruitment documents, and the final study summary disseminated to participants).

Results

Of 48 patients invited, 17 agreed to take part in interviews (mean time since surgery 3.11 years, range four months to nine years), although two others (spouses of existing participants) were opportunistically recruited as the research was ongoing. Interviews lasted between 44 and 110

FUNDED BY NIHR | National Institute for Health Research
For peer review only - http://bmjopen.bmj.com/site/about/guidelines.xhtml
UOB Confidential
For peer review only

minutes. Twelve of the 19 participants were female, and the mean age was 51.1 years. All reported their ethnicity to be 'White British', and 17 had already undergone surgery **(Table 1)**. The analysis presented draws on interview data from the 17 participants that had undergone surgery.

Bariatric surgery was a life-changing journey for participants, impacting on several different areas of their lives. The overarching concepts of 'normality' and 'ambivalence' emerged from analysis of data on patients' experiences of adapting to life after surgery **(Figure 1)**. Analysis of data relating to experiences of follow-up care was conducted separately and characterised by two concepts – 'abandonment' and 'isolation' **(Figure 2)**. Results are presented according to overarching concept with participant quotes used to support the description of each concept.

Adapting to life after surgery – normality and ambivalence

Throughout several areas of their lives, participants were striving to be more "normal" after bariatric surgery. This related to different aspects of their lives categorised as physical health, psychological health, eating patterns and hunger, body image, weight, and social functioning **(Figure 1)**. Participants experienced many positive changes that undeniably brought them closer to their idea of normality. However, participants also described things that did not change, for which they still felt abnormal. Some also experienced changes perceived as negative or difficult to deal with, which made them feel more abnormal and required a process of adjustment. This was acknowledged as a "trade-off", or the "price to pay" (P08) for the benefits gained. The complexity of the changes experienced highlighted the ambivalence of living with the results of bariatric surgery. Despite the challenges, all participants felt the surgery was a good decision: "I don't regret it for a minute. Despite all the complications and issues." (P14)

Normality

All participants reported an improvement in activity and mobility levels and/or their ability to carry out 'normal' activities of daily living following surgery: "I'm more mobile, I can tie my shoelaces,

FUNDED BY NIHR | National Institute for Health Research

For peer review only - http://bmjopen.bmj.com/site/about/guidelines.xhtml

For peer review only

UOB Confidential

shower properly…my life has changed for the better." (P10). Participants also reported several positive changes related to physical and psychological health including a reduction in medications required (e.g. for diabetes), an improvement in physical symptoms (such as joint pain), self-confidence, and psychological well-being: "I feel healthier mentally in my head, like I want to get out there." (P09).

Some participants described an improved or more 'normal' relationship with food after surgery, whereby they had retrained their mind to focus on "eating more sensibly" rather than thinking they were "on a diet" (P11). Others experienced no real change to their relationship with food, feeling as though they still had to be "on a permanent diet" (P19), or continued to use food as way of coping with difficult emotions which remained: "I still have an awkward relationship with food…still have the same demons…I probably rely on food to deal with certain emotions" (P14).

All 17 of the participants had lost a large amount of weight since having surgery, however, eight had re-gained some of this weight. Participants reported feeling distressed by this as they did not want to return to the way they were: "That was a real horrendous thing for me to see my weight go up a bit after all I'd gone through to get it down..." (P07). However, a couple described being reassured by health professionals that it was normal to experience some weight re-gain. The majority related their weight re-gain to a gradual increase in appetite and/or portion sizes over time (which had initially decreased after surgery), and a feeling that the surgery was not as effective as it had been: "I don't seem to be getting the urge to stop quicker, like I did before" (P18).

The majority of participants reported developing loose-hanging excess skin following their massive weight loss, which challenged their sense of normality. Although they were pleased to be a more 'normal' size, some felt ashamed of how abnormal their body looked without clothes on. Skin removal surgery was a costly option, so some had learned to live with the excess skin; however, a few found the excess skin to be particularly problematic, impacting on their mental health and

For peer review only - http://bmjopen.bmj.com/site/about/guidelines.xhtml

FUNDED BY NIHR National Institute for Health Research

UOB Confidential

relationships: "My husband doesn't like the excess skin…and that's one of the reasons why I must do something about it, because…I know I look like a bag of s**t" (P12).

Ambivalence

Although improvements to existing health problems were important benefits of the surgery, five participants reported developing new health problems post-surgery, including micronutrient deficiencies, menstrual problems, brittle bones, low blood pressure and cardiac issues: "…you give up one set of health implications but you get given another set in its place..." (P07). Some participants still suffered with several food intolerances and/or frequent gastrointestinal symptoms many years after surgery, which they reported resulted in a poorly balanced diet: "I can't eat bread or meat…That's one of the small prices I have to pay…my intake of food is nowhere near balanced…" (P08).

Difficulties were described in developing new coping strategies to replace food, which had previously been a "comfort blanket": "…all your insides are different but your brain…no different whatsoever…that for me was the hardest thing to adjust to, because my brain was still telling my stomach I was hungry but obviously I couldn't [eat]..." (P03). One patient described developing an alcohol dependency post-surgery (which they had eventually overcome), and two participants mentioned the need for more psychological input to help with their adjustment following surgery: "There was no formal counselling…and that might be a good idea to find out why we eat so much, why are we addicted to food…" (P04).

Ambivalence was also evident in participants' experiences of social functioning and stigma. Participants reported receiving positive attention due to their weight loss: "…people tell you 'you look brilliant'…that is the good side of it" (P17). For some, however, this led to mixed emotions at the revelation of "how negative people saw you before" (P07). Others described receiving less negative attention and feeling less socially stigmatised due to their obesity: "I can walk down the road now and not get such the bad looks as I used to." (P04). However, a number of participants had

FUNDED BY NIHR | National Institute for Health Research

For peer review only - http://bmjopen.bmj.com/site/about/guidelines.xhtml

UOB Confidential

experienced a new type of social stigma at having taken the "easy way out" (P02) by having surgery (e.g. not achieved weight loss through the 'normal' means). Some were ashamed to tell others they had undergone surgery for fear of this reaction.

Social and family eating situations could also cause anxiety for some due to attracting attention for only eating very small amounts, or unpleasant and embarrassing gastrointestinal symptoms which could arise when eating. For some this had remained an issue several years following surgery causing disruption to relationships: "It disrupts life because I can be eating and whether it's the wrong food, a mouthful too much…I've got to go out and she can hear me retching, and it puts her off her food" (P08). Others were able to adapt or reported their social life had "come back" (P10) gradually as food tolerance improved.

Experiences of follow-up care – abandonment and isolation

Participants explained that follow-up care received after surgery was mainly provided by the specialist bariatric surgery team (although what this entailed was highly variable), with little support from their GPs (general practitioners). Only a few participants described feeling well-supported overall, and all of these had undergone their surgery less than two years previously. However, most described at least one aspect of follow-up care which they found helpful. These included: 1) the routine monitoring of certain measures (e.g. weight, nutritional blood tests); 2) the availability of one key health professional (generally a specialist dietitian or nurse), who was easy to contact on an ad-hoc basis; 3) the ability to contact the bariatric team using a range of contact options (e.g. telephone, email); 4) good communication between team members; and 5) continuity of care (e.g. being able to see the same professionals at every appointment) **(Table 2)**.

Overall, however, there was a sense of abandonment and isolation in participants' accounts of follow-up care. This related to their experiences of post-operative support from the specialist team, primary care professionals, and peer support groups **(Figure 2)**. Participants felt that health professionals didn't always appreciate the long-term implications of life after surgery, or even if they

FUNDED BY NIHR | National Institute for Health Research

For peer review only - http://bmjopen.bmj.com/site/about/guidelines.xhtml

UOB Confidential

did, services were not set up to support them adequately: “It happened eight years ago so no one thinks you may have any hang-ups, issues, concerns about it...the implications of the changes it makes people don't really appreciate, it’s an old record, old news.” (P07).

Abandonment

Some participants felt that problems or complications they experienced following surgery were ignored or not dealt with properly, or there was a lack of clarity of who to go to if they experienced problems. P07, for example, felt her post-operative problems were dismissed by the specialist team, and that she “was upsetting someone’s figures by having complications”. P12 experienced a problem with one of her surgical wounds which wouldn’t heal and wasn’t sure who to go to about it. She felt “quite abandoned” and dealt with it mainly on her own. Abandonment also related to the feeling they had been given inadequate information or guidance about life following surgery: “They give you loads of information about what to do in the first six weeks and then there’s nothing…” (P04).

Abandonment was also evident in accounts of support only being provided when patients themselves initiated contact: “I feel that as long as you didn’t contact them then you will be left alone…” (P15). Concerns were raised for others whom they perceived less likely to seek help proactively: “…these people aren’t coming forward to explain that they’re having problems because they don’t want to feel like a failure…” (P09). P18 expressed disappointment that he had not been sent any appointments post-operatively and felt he had been left “in limbo” to “get on with it” himself. He had not asked for help and was under the impression that it would only be appropriate to contact the team if you were having complications: “…obviously if I was in excruciating pain from the operation I suppose, I could have gone back…” (P18).

Most participants also reported feeling abandoned by their GPs who were not usually supportive of them having undergone bariatric surgery and did not “fully appreciate the struggles that you have” (P14) in the long-term. However, a minority of participants described feeling well-supported by their GPs who recognised the long-term health benefits of bariatric surgery: “…with being my dad’s

FUNDED BY NIHR | National Institute for Health Research

For peer review only - http://bmjopen.bmj.com/site/about/guidelines.xhtml

UOB Confidential

doctor, he sees that hopefully I won't have the same problems…he's done everything he can to help me…" (P05).

Isolation

Several participants did not live locally to the hospital where the specialist team were located. This presented a barrier to accessing follow-up care, which some felt could contribute to feelings of isolation: "From this side of the county it's (hospital) extremely difficult to get to…I can understand an awful lot of people thinking "if I ring [hospital] they're going to say come over and see me and that is so difficult to get to…I won't bother" (P15). Equally participants described how local primary care services were unable to support them compounding their feelings of isolation: "Unless they've (GP surgery) read my notes they don't even know I've got one (a gastric band)" (P04), and " They (GP surgery) were very much like 'it's a secondary care issue, it's not primary care" (P07).

Isolation was also apparent in participants' experiences of bariatric surgery peer support groups. Although not part of medical care, these represented an important source of support. These groups were typically run by patient volunteers, with limited or no input from health professionals. Some participants had access to these groups in their local areas, whereas others did not. Those unable to access a group felt this contributed to their sense of isolation post-surgery: "…there's meetings where you can meet other people who've had the [gastric] band…but there's no local ones for me…if people said, 'If you do eat it, it's going to hurt but it will go, and this is the reason it's hurting,' then I could have dealt with it a little bit better." (P17). Those that had accessed these groups described variable experiences. Some found them supportive, for example P01 who continued to attend several years post-surgery, whereas others had negative experiences and felt quite isolated from other members. P19, for example, had disengaged from her local group which she described as being very "cliquey" with members using the group mainly to emphasize negative experiences or "how to cheat the band". Many felt that peer support groups including "a chairman" (P15)

For peer review only

FUNDED BY NIHR | National Institute for Health Research

UOB Confidential

knowledgeable in the results of bariatric surgery should be part of routine clinical care to improve accessibility of peer support and ensure consistency of information discussed.

Discussion

This qualitative study found that bariatric surgery impacted participants' physical and psychological health, eating behaviours, weight, and social functioning. The overarching concepts of normality and ambivalence illustrated their lived experience following bariatric surgery. Normality was evidenced through participants' relief at feeling more normal in some ways (e.g. improved ability to undertake daily activities), yet feeling less normal in other areas, including the development of excess skin and difficulties eating 'normally' in social situations. Although participants experienced many positive health changes, they also experienced changes which were negative or difficult to adapt to, such as an inability to rely on emotional eating as an entrenched coping mechanism, perceived bodily deformity as a result of excess skin, and the destabilisation of important relationships. These complexities highlight the ambivalence of living with the outcomes of bariatric surgery. In coping with these changes, participants received varying levels of care from specialist health professionals and GPs. Although there were some positive experiences, 'abandonment' and 'isolation' characterised most follow-up care experiences. This included feeling unsupported with post-surgery problems (other than serious complications), lack of guidance with long-term lifestyle changes, lack of understanding from GPs, and limited peer support. However, all participants felt that undergoing the surgery was a good decision despite the difficulties. These findings are important in helping to define future follow-up care packages to better address the complex changes experienced after bariatric surgery.

Our findings are consistent with previous qualitative research on patient experiences of living with outcomes of bariatric surgery which depicted the complexities on patients' sense of normality and the 'give and take' or ambivalent nature of the changes experienced.[10 41-43] This study strengthens

FUNDED BY NIHR | National Institute for Health Research
For peer review only - http://bmjopen.bmj.com/site/about/guidelines.xhtml
UOB Confidential

the evidence for the individual and nuanced nature of how bariatric surgery changes people's relationship with food in different ways, and changes over time, indicating the need for individualised dietary and psychological support at different time-points.[10 28 41 43 44] The importance placed by participants on the social impact of bariatric surgery was also noted in a recent UK study by Graham *et al*.[45] These issues, including difficulties with social and family eating should be given more attention in follow-up care. Our study confirms previous qualitative findings on the importance of continuity of care,[19] the ability to access professional advice (often from the specialist dietitian) between appointments via telephone or email,[31] the lack of psychological support after surgery,[19 28-30 32 33 36 46] and the need for moderation in patient support groups.[33 34] Previous studies have related patients' views that GPs were not equipped to adequately support them post-surgery.[19 30 31 47] This was also evident in our study with most participants describing negative experiences with GPs in relation to bariatric surgery, and feeling they were unable to offer adequate support. Despite this, several participants would have preferred to access support locally due to living remotely.

Our study expanded the findings on patient experiences of bariatric surgery follow-up care as being characterised by feelings of abandonment and isolation, with views that services were not set up to support long-term issues. Abandonment was also evident in a study by Jumbe & Meyrick who described a "post-surgical cliff" with patients receiving intensive support prior to bariatric surgery and then feeling abandoned after surgery.[36] Similar to our study, they described how post-operative support was reliant on patient-initiated contact. Previous research with people living with obesity suggests they may delay or avoid seeking healthcare due to societal and medical stigmas.[48 49] This has also been reported by Throsby who conducted a UK-based ethnographic study within a surgical weight management clinic.[50] She described examples of patients struggling with their eating habits and weight post-surgery, and the shame they felt at doing 'badly' after undergoing publicly-funded surgery. The author argued that this "moral weight" could lead to patients not seeking help when most needed.[50] Similarly, feelings of shame and failure at not having met the perceived post-

FUNDED BY NIHR | National Institute for Health Research

For peer review only - http://bmjopen.bmj.com/site/about/guidelines.xhtml

UOB Confidential

operative expectations was one reason cited by Australian patients for non-attendance in bariatric surgery aftercare.[30]

The main strength of this research is that a detailed qualitative approach to data collection was used, whereby participants were given the time and flexibility to relate their own experiences in terms that were relevant for them. A rigorous approach to analysis was undertaken, including independent coding of initial transcripts by three researchers, and discussion and agreement of emergent themes throughout analysis with at least one other qualitative researcher. A limitation of this study is the lack of ethnic diversity represented within the sample. Low numbers of people from ethnic minority groups undergo bariatric surgery in the UK (1303 between 2011-2013, 7.7% of total procedures), making it difficult to identify eligible people for qualitative studies.[14] A strength of this research is that we were able to over-represent male participants within our sample (41% of the 17 post-operative participants compared with 24% who undergo bariatric surgery nationally), which has been a limitation of previous qualitative studies in this area.[14 28 30 32 34-36] An additional strength was the inclusion of a clinically diverse group of patients who had undergone all three main types of bariatric procedures in the UK and who were at a broad range of timepoints post-surgery. Participants were also recruited from two UK centres with different follow-up programmes and health professional teams. It is not known, however, whether similar themes would be found with participants in other centres. The findings relating to follow-up care may be less generalisable to healthcare systems with different service pathways and funding structures.

Taken together with previous literature, our findings highlight that current bariatric surgery follow-up care provision is not often aligned with patient need. Patients highlighted the need for a flexible and long-term approach to follow-up care from a multi-disciplinary health professional team. This includes both routine and open appointments, moderated peer support groups, and different methods of contact (e.g. telephone, online in addition to face-to-face). These recommendations are also in accordance with the recently published 2019 UK psychological guidelines for bariatric surgery

FUNDED BY NIHR | National Institute for Health Research

For peer review only - http://bmjopen.bmj.com/site/about/guidelines.xhtml

UOB Confidential

For peer review only

which recommend a flexible and individualised approach to post-operative psychological support, including routine screening at 6-9 months post-surgery to identify support needs.[51] In addition to individual dietary and psychological support, services should consider how to better support patients in developing strategies to cope with family and social difficulties post-surgery. This may include actively engaging family and close friends in pre-operative preparation and/or post-operative interventions. Future research is needed to define and evaluate an effective and acceptable follow-up care package that could be consistently applied across bariatric surgery centres. This may include the optimal systems or pathways to identify and support those who need the most help but are the least likely to seek it, ways of engaging family and social support, and delivering moderated peer support groups. The relative merits of delivering follow-up care in specialist or community-based health services or how it might be shared between the two should also be investigated.

Author contributions

KDC led the study design, data collection and data analysis as part of her PhD research, and drafted this manuscript. AOS, FM and JMB were KDC's PhD supervisors and advised on study design, data collection and analysis, and provided comments on this manuscript. JLD advised and contributed to data analysis and provided comments on this manuscript. All authors approved the final submitted manuscript.

Acknowledgements

The authors would like to thank the patient research partners who advised on this research for their valuable input, as well as the study participants who gave up their time to take part in the research and the health professionals at the centres who helped with study recruitment.

For peer review only

FUNDED BY NIHR | National Institute for Health Research

UOB Confidential

Data sharing

Anonymised participant data can be made available on reasonable request to the corresponding author at karen.coulman@bristol.ac.uk.

References

1. World Health Organisation. Obesity and overweight. 16 February 2018. https://www.who.int/en/news-room/fact-sheets/detail/obesity-and-overweight. Date accessed 15.10.19.
2. Must A, Spadano J, Coakley EH, et al. The disease burden associated with overweight and obesity. *JAMA* 1999;282(16):1523-29.
3. Wang Y, McPherson K, Marsh T, et al. Health and economic burden of the projected obesity trends in the USA and the UK. *Lancet* 2011;378(9793):815-25.
4. Dietz WH, Baur LA, Hall K, et al. Management of obesity: improvement of health-care training and systems for prevention and care. *Lancet* 2015;385(9986):2521-33.
5. National Institute for Health and Care Excellence. Obesity: identification, assessment and management. Clinical Guideline [CG189]. London, 2014. Available from: https://www.nice.org.uk/guidance/cg189. Date accessed: 15.10.19
6. Collins J, Meng C, Eng A. Psychological Impact of Severe Obesity. *Current Obes Rep* 2016;5(4):435-40.
7. Kolotkin RL, Crosby RD, Williams GR. Health-related quality of life varies among obese subgroups. *Obes Res* 2002;10(8):748-56.
8. Owen-Smith A, Donovan J, Coast J. "Vicious circles": the development of morbid obesity. *Qual Health Res* 2014;24(9):1212-20.
9. Wu YK, Berry DC. Impact of weight stigma on physiological and psychological health outcomes for overweight and obese adults: A systematic review. *J Adv Nurs* 2018;74(5):1030-42.
10. Coulman KD, MacKichan F, Blazeby JM, et al. Patient experiences of outcomes of bariatric surgery: a systematic review and qualitative synthesis. *Obes Rev* 2017;18(5):547-59.
11. Coulman KD, Abdelrahman T, Owen-Smith A, et al. Patient-reported outcomes in bariatric surgery: a systematic review of standards of reporting. *Obes Rev* 2013;14(9):707-20.
12. Colquitt Jill L, Pickett K, Loveman E, et al. Surgery for weight loss in adults. *Cochrane Database Syst Rev* 2014 Aug 8;(8):CD003641. doi: 10.1002/14651858.CD003641.pub4
13. O'Brien PE, MacDonald L, Anderson M, et al. Long-term outcomes after bariatric surgery: fifteen-year follow-up of adjustable gastric banding and a systematic review of the bariatric surgical literature. *Ann Surg* 2013;257(1):87-94.
14. Welbourn R, Small P, Finlay I, et al. The United Kingdom National Bariatric Surgery Registry - 2nd Registry Report. Henley-on-Thames: Dendrite Clinical Systems Ltd, 2014.

For peer review only - http://bmjopen.bmj.com/site/about/guidelines.xhtml
FUNDED BY NIHR National Institute for Health Research
UOB Confidential
For peer review only

15. Welbourn R, Hollyman M, Kinsman R, et al. Bariatric Surgery Worldwide: Baseline Demographic Description and One-Year Outcomes from the Fourth IFSO Global Registry Report 2018. *Obes Surg* 2019;29(3):782-95.
16. Miras AD, le Roux CW. Mechanisms underlying weight loss after bariatric surgery. *Nat Rev Gastroenterol Hepatol* 2013;10(10):575-84.
17. Rogers CA, Reeves BC, Byrne J, et al. Adaptation of the By-Band randomized clinical trial to By-Band-Sleeve to include a new intervention and maintain relevance of the study to practice. *Br J Surg* 2017;104(9):1207-14.
18. Lindekilde N, Gladstone BP, Lubeck M, et al. The impact of bariatric surgery on quality of life: a systematic review and meta-analysis. *Obes Rev* 2015;16(8):639-51.
19. Parretti HM, Hughes CA, Jones LL. 'The rollercoaster of follow-up care' after bariatric surgery: a rapid review and qualitative synthesis. *Obes Rev* 2019;20(1):88-107.
20. Spaniolas K, Kasten KR, Celio A, et al. Postoperative Follow-up After Bariatric Surgery: Effect on Weight Loss. *Obes Surg* 2016;26(4):900-3.
21. National Confidential Enquiry into Patient Outcome and Death. Too Lean a Service? A review of the care of patients who underwent bariatric surgery. 2012. https://www.ncepod.org.uk/2012report2/downloads/BS_fullreport.pdf. Date accessed: 15.10.19.
22. Mechanick JI, Youdim A, Jones DB, et al. Clinical Practice Guidelines for the Perioperative Nutritional, Metabolic, and Nonsurgical Support of the Bariatric Surgery Patient—2013 Update: Cosponsored by American Association of Clinical Endocrinologists, The Obesity Society, and American Society for Metabolic & Bariatric Surgery. *Surg Obes Relat Dis* 2013;9(2):159-91.
23. Coulman KD, Hopkins J, Brookes ST, et al. A Core Outcome Set for the Benefits and Adverse Events of Bariatric and Metabolic Surgery: The BARIACT Project. *PLoS Med* 2016;13(11):e1002187. doi: 10.1371/journal.pmed.1002187
24. Richards T, Montori VM, Godlee F, et al. Let the patient revolution begin. *BMJ* 2013;346:f2614. doi: 10.1136/bmj.f2614
25. Pope C, Mays N. Qualitative Methods in Health Research. In: Pope C, Mays N, eds. Qualitative Research in Health Care, Third edition. Malden, Massachusetts: Blackwell Publishing Ltd 2006:12-20.
26. Carter S, Henderson L. Approaches to qualitative data collection in social science. In: Bowling A, Ebrahim S, eds. Handbook of Health Research Methods: Investigation, Measurement and Analysis. Maidenhead, Berkshire: Open University Press 2005:215-29.
27. Snape D, Spencer L. The Foundations of Qualitative Research. In: Ritchie J, Lewis J, eds. Qualitative Research Practice: A Guide for Social Science Students and Researchers. London: SAGE Publications Ltd 2003:1-23.
28. Ogden J, Avenell S, Ellis G. Negotiating control: Patients' experiences of unsuccessful weight-loss surgery. *Psychol Health* 2011;26(7):949-64.
29. Ivezaj V, Saules KK, Wiedemann AA. "I didn't see this coming.": why are postbariatric patients in substance abuse treatment? Patients' perceptions of etiology and future recommendations. *Obes Surg* 2012;22(8):1308-14.
30. Moroshko I, Brennan L, Warren N, et al. Patients' Perspectives on Laparoscopic Adjustable Gastric Banding (LAGB) Aftercare Attendance: Qualitative Assessment. *Obes Surg* 2014;24(2):266-75.
31. Aarts MA, Sivapalan N, Nikzad SE, et al. Optimizing Bariatric Surgery Multidisciplinary Follow-up: a Focus on Patient-Centered Care. *Obes Surg* 2017;27(3):730-36.
32. Lauti M, Stevenson S, Hill AG, et al. Patient Perspectives About Follow-Up Care and Weight Regain Following Sleeve Gastrectomy. *Obes Surg* 2016;26(11):2724-31.
33. Sharman M, Hensher M, Wilkinson S, et al. What are the support experiences and needs of patients who have received bariatric surgery? *Health Expect* 2017;20(1):35-46.

FUNDED BY NIHR National Institute for Health Research

For peer review only - http://bmjopen.bmj.com/site/about/guidelines.xhtml

UOB Confidential

34. Jones L, Cleator J, Yorke J. Maintaining weight loss after bariatric surgery: when the spectator role is no longer enough. *Clin Obes* 2016;6(4):249-58.
35. Pfeil M. Patients' perception of the nursing contribution to gastric band surgery. *Br J Nurs* 2014;23(2):95-9
36. Jumbe S, Meyrick J. Contrasting Views of the Post-bariatric Surgery Experience between Patients and their Practitioners: a Qualitative Study. *Obes Surg* 2018;28(8):2447-56.
37. Glaser B, Strauss A. The discovery of grounded theory: Strategies for qualitative research. Chicago: Aldine 1967.
38. Legard R, Keegan J, Ward K. In-depth Interviews. In: Ritchie J, Lewis J, eds. Qualitative Research Practice: A Guide for Social Science Students and Researchers. London: SAGE Publications Ltd 2003:138-69.
39. Corbin J, Strauss A. Chapter 8: Analysing data for concepts. Basics of Qualitative Research: Techniques and Procedures for Developing Grounded Theory, 3rd edition. Thousand Oaks, CA: SAGE Publications, Inc. 2008:159-94.
40. NVivo 10. Melbourne: QSR International Pty Ltd., 2013.
41. Warholm C, Marie Oien A, Raheim M. The ambivalence of losing weight after bariatric surgery. *Int J Qual Stud Health Well-being* 2014;9:22876.
42. Natvik E, Gjengedal E, Raheim M. Totally changed, yet still the same: patients' lived experiences 5 years beyond bariatric surgery. *Qual Health Res* 2013;23(9):1202-14.
43. Meana M, Ricciardi L. Obesity surgery: Stories of altered lives. Reno, Nevada: University of Nevada Press 2008.
44. Engstrom M, Forsberg A. Wishing for deburdening through a sustainable control after bariatric surgery. *Int J Qual Stud Health Well-being* 2011;6(1).
45. Graham Y, Hayes C, Small PK, et al. Patient experiences of adjusting to life in the first 2 years after bariatric surgery: a qualitative study. *Clin Obes* 2017;7(5):323-35.
46. Stolzenberger KM, Meaney CA, Marteka P, et al. Long-term quality of life following bariatric surgery: A descriptive study. *Bariatr Surg Pract Patient Care* 2013;8(1):29-38.
47. Jose K, Venn A, Nelson M, et al. A qualitative study of the role of Australian general practitioners in the surgical management of obesity. *Clin Obes* 2017;7(4):231-38.
48. Drury CA, Louis M. Exploring the association between body weight, stigma of obesity, and health care avoidance. *J Am Acad Nurse Pract* 2002;14(12):554-61.
49. Olson CL, Schumaker HD, Yawn BP. Overweight women delay medical care. *Arch Fam Med* 1994;3(10):888-92.
50. Throsby K. Obesity surgery and the management of excess: exploring the body multiple. *Sociol Health Illn* 2012;34(1):1-15.
51. Ogden J, Ratcliffe D, Snowdon-Carr V. British Obesity Metabolic Surgery Society endorsed guidelines for psychological support pre- and post-bariatric surgery. *Clin Obes* 2019;9(6):e12339.

For peer review only

FUNDED BY NIHR | National Institute for Health Research
For peer review only - http://bmjopen.bmj.com/site/about/guidelines.xhtml
UOB Confidential

Table 1: Characteristics of participants

Participant	Gender	Age range (years)	Marital status	Employment status	Type of surgery	Time since surgery (years)
P01	Female	60-70	Married	Retired	RYGB	>5
P02	Female	50-60	Married	Unemployed	RYGB	<1
P03	Female	30-40	Married	Employed*	RYGB	1-2
P04	Female	60-70	Married	Retired	AGB	>5
P05	Male	40-50	Married	Employed	RYGB	<1
P06	Female	30-40	Married	Employed	Awaiting surgery	N/A
P07	Female	40-50	Married	Employed	RYGB	>5
P08	Male	60-70	Married	Employed	AGB	>5
P09	Female	40-50	Married	Unemployed	SG	1-2
P10	Male	30-40	Co-habiting	Self-employed	SG	2-5
P11	Female	40-50	Married	Employed	SG	<1
P12	Female	50-60	Married	Self-employed	SG	1-2
P13	Male	50-60	Widowed	Employed	RYGB	<1
P14	Female	40-50	Married	Employed	AGB & RYGB	>5
P15	Male	60-70	Married	Retired	RYGB	1-2
P16	Female	60-70	Married	Retired	Awaiting surgery	N/A
P17	Male	40-50	Married	Employed	AGB	2-5
P18	Male	50-60	Co-habiting	Employed	AGB	1-2
P19	Female	30-40	Separated	Employed	AGB	1-2

AGB=Adjustable gastric band, RYGB=Roux-en-Y gastric bypass, SG=Sleeve gastrectomy

***'Employed' status includes those employed both full-time and part-time**

For peer review only

FUNDED BY NIHR National Institute for Health Research

For peer review only - http://bmjopen.bmj.com/site/about/guidelines.xhtml

UOB Confidential

1 **Table 2: Participant quotes to support positive experiences of follow-up care**

Positive aspects of care	**Quotes**
Routine monitoring of certain measures	"It was good having my bloods done so I could check what my levels were like, that was quite useful for me…routine monitoring was good." (P07)
The availability of a key health professional; Ability to contact the team using a range of contact options	"If I couldn't get hold of her (dietitian) straight away on the phone I'd send an email and it would either be answered the same day or the next day." (P09)
Good communication between team members	"It's quite a tight little team….you might not necessarily speak to the best person, but they will come together in their meeting and you'll get the best outcome." (P19)
Continuity of care	"You didn't see twenty different people. It was 'the team'…the same faces…I like that. I don't want to see somebody who's different don't know you…" (P08)
Overall positive view of care	"The follow-up care I've had has just been 110%, if I've had a problem I would ring and…I would get an appointment…Someone has always been there for me…" (P01)

For peer review only

FUNDED BY NIHR National Institute for Health Research

UOB Confidential

Figure legends

Figure 1: Concepts and categories illustrating the adaptation to life after bariatric surgery including an example of supporting themes for one category

Figure 2: Concepts and categories illustrating the experiences of follow-up care after bariatric surgery including supporting themes

For peer review only

FUNDED BY NIHR | National Institute for Health Research

UOB Confidential

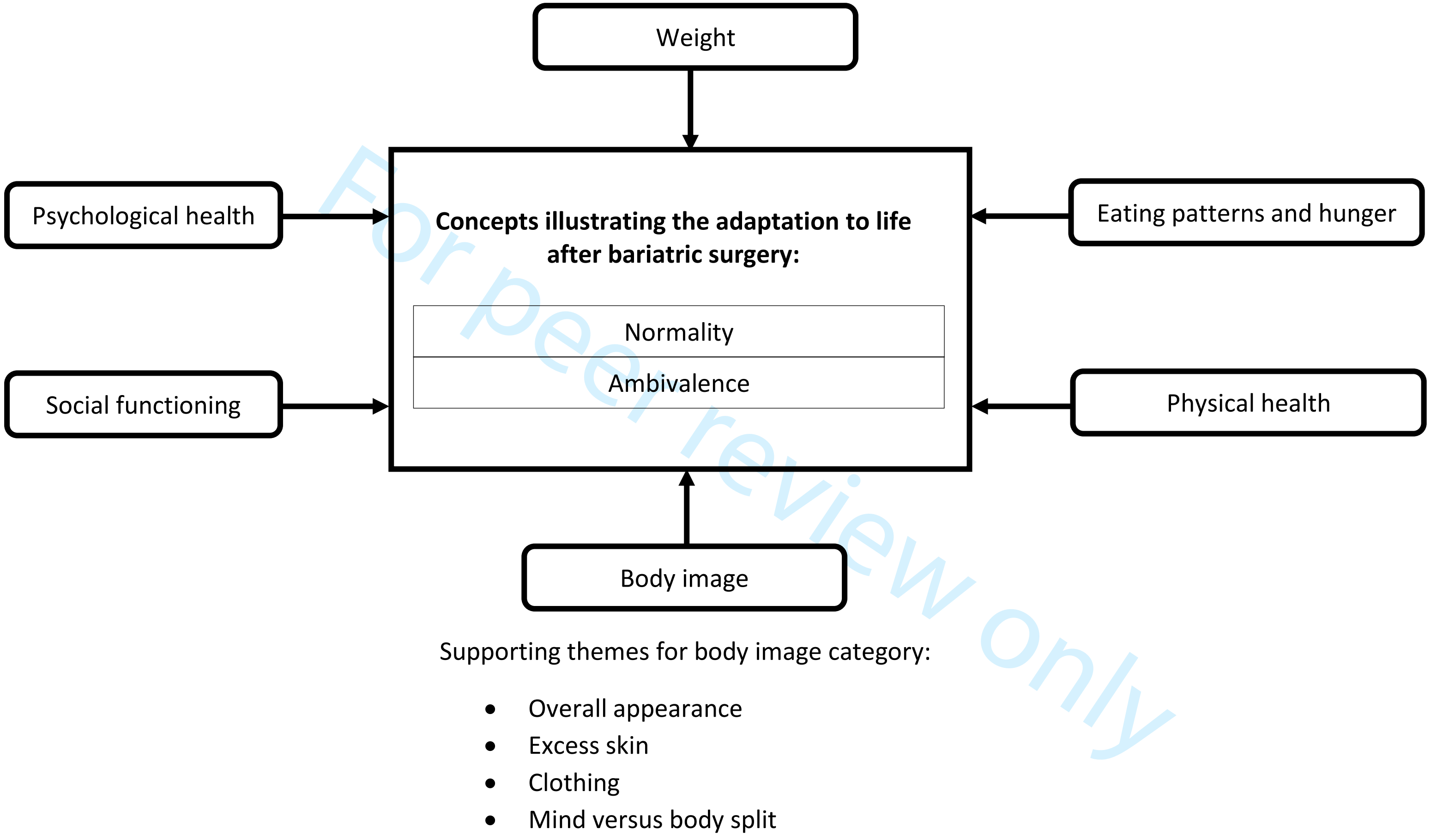

Figure 1: Concepts and categories illustrating the adaptation to life after bariatric surgery including an example of supporting themes for one category

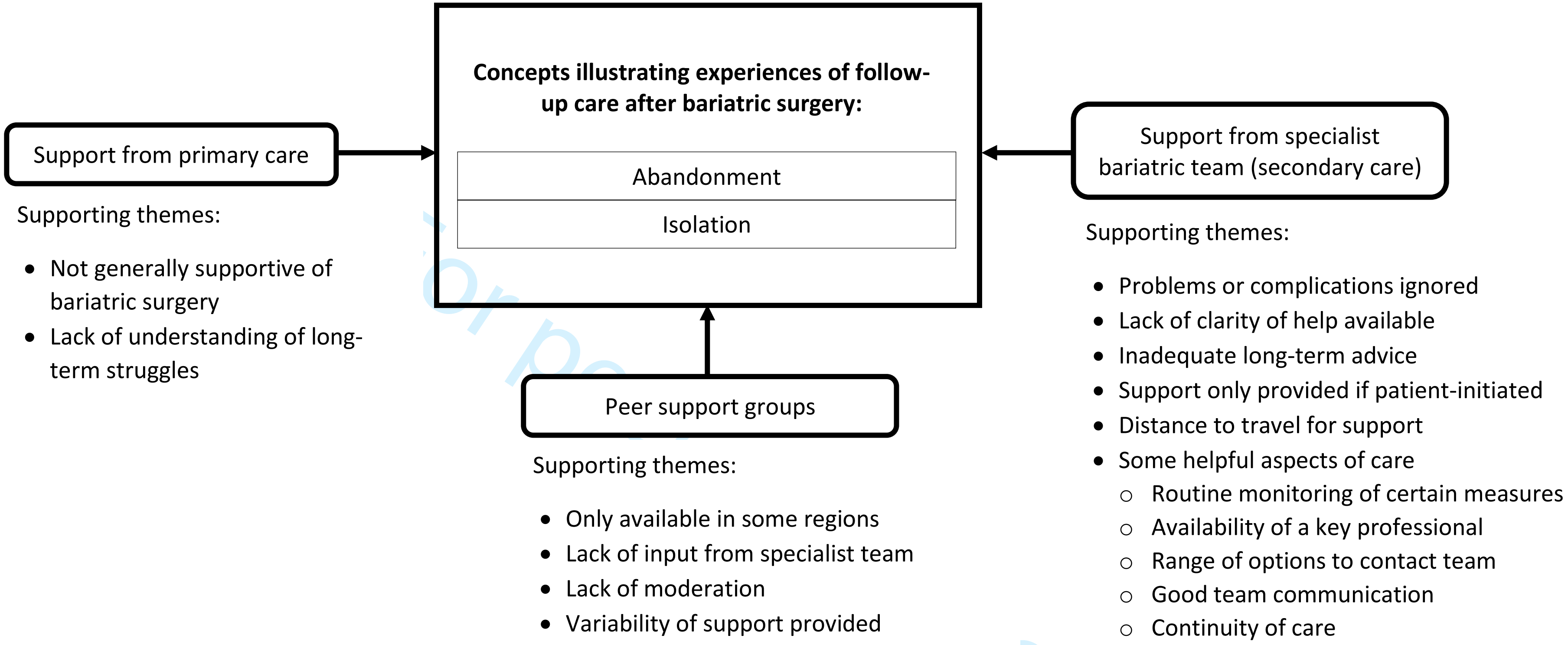

Figure 2: Concepts and categories illustrating the experiences of follow-up care after bariatric surgery including supporting themes

Study Protocol version 2.0 (7/7/14)

Document S1 (Study protocol)

The patient perspective of living with surgery for morbid obesity: Creating a patient 'core' outcome set, and investigating ways to improve follow-up care

Chief Investigator

Karen D Coulman, NIHR Doctoral Research Fellow, School of Social and Community Medicine, Unversity of Bristol

Supervisors

Dr Amanda Owen-Smith, School of Social and Community Medicine, Unversity of Bristol

Professor Jane M Blazeby, School of Social and Community Medicine, Unversity of Bristol

Funding

National Institute of Health Research (NIHR) Doctoral Research Fellowship award

For peer review only

For peer review only - http://bmjopen.bmj.com/site/about/guidelines.xhtml

Lay Summary

Surgery is increasingly being used as a treatment for obesity. Studies report many different outcomes, often measured in different ways, and little is known about what outcomes are important to patients. This study aims to explore the outcomes that are important to patients and to use this information to develop a short list of the most important outcomes to health professionals and patients (a core outcome set), which can be used to evaluate surgical treatments for obesity. In addition, this study will investigate patients' experiences of obesity surgery to provide recommendations for how post-operative support can be improved in the NHS. This will be achieved by interviewing obesity surgery patients to determine whether published outcomes reflect those outcomes patients themselves consider important, and to obtain views on living with surgery and ways to improve post-operative support. A list of patient-centred outcomes will be created from the literature and the interviews which will be added to a list of 'medical' outcomes of obesity surgery. This long list of outcomes will be reduced into a "core" set of outcomes, using a scientific process to reach agreement, which involves 2 rounds of questionnaires completed by health professionals and patients. The final core outcome set will be agreed in separate consensus meetings with a small group of health professionals and patients. This core outcome set will be useful to researchers in choosing outcomes for research trials that are important to both health professionals and patients. Use of the core outcome set would also improve the overall quality of reporting in trials. This research will also be useful for health professionals to ensure health services are designed based on patient priorities.

For peer review only

For peer review only - http://bmjopen.bmj.com/site/about/guidelines.xhtml

Background

Obesity surgery

The NHS Information Centre has released figures showing that just over one quarter of the adult population of England was obese in 2010, an increase from 13-16% of the population in 1993[1]. It is predicted that one third of adults in England will be obese by 2015 and more than 700 million adults will be obese worldwide by 2015[1;2]. Obesity is associated with an increased risk of type 2 diabetes, heart disease, cancer, reduced quality of life and premature death[1;3]. As such, obesity is a major public health concern. Various treatments for obesity exist including lifestyle modifications, pharmacotherapy, and more recently, surgical interventions. Obesity surgery operations have rapidly increased in the UK, and worldwide, with the most common operations being the adjustable gastric band and the Roux-en-Y gastric bypass[1;4]. In 2009, a Health Technology Assessment report (including a Cochrane systematic review) concluded that obesity surgery is more clinically effective than other treatments for obesity in terms of weight loss, improvement of co-morbidities such as type 2 diabetes and hypertension, and is also cost effective[5].

The patient perspective of outcomes of obesity surgery

Although clinical outcomes are important to measure, patient-reported outcome (PRO) measures, such as measures of health-related quality of life (HRQL), provide a means for capturing how a patient feels about their health or condition[6]. The Food and Drug Administration (FDA) has published guidance on the development of PRO measures to support claims of treatment benefit[6]. PRO measures should be developed based on rigorous qualitative research with patients to ensure content validity (authenticity to patients)[6;7]. Despite this, the development of PRO measures is variable and not always transparent[7].

The lead researcher has recently undertaken a systematic review of PROs in obesity surgery studies, which identified 68 different validated questionnaires used to assess PROs amongst the 86 identified studies [8]. Due to the heterogeneity of the items and scales within the questionnaires used amongst the various studies, a meta-analysis to determine the effect of obesity surgery on PROs was unable to be undertaken. The lead researcher then looked in further depth at the development of those questionnaires which were classified as obesity-specific (19 questionnaires). Although 14 (74%) reported to have involved patients in questionnaire development, only 3 (16%) specified that obesity surgery patients were involved. Only 6 (32%) specified that qualitative interviews/focus groups were used to obtain patient views and none specified that a qualitative analysis of resulting data was undertaken. Thus, it is not certain whether the majority of PRO questionnaires being used to assess obesity surgery are grounded in patient views.

Although well-developed PRO measures can provide valuable information about the patient's perspective of outcomes of treatment, primary qualitative studies can also yield rich information; however, these types of studies are rarely included in Cochrane reviews of quantitative evidence. The lead researcher is currently undertaking a review of qualitative research studies where the patient perspective of obesity surgery was sought. Although qualitative studies have focused on patient experiences of surgery, none have explicitly investigated patients' views of important outcomes of obesity surgery.

There is clearly more work needed to clarify the outcomes of obesity surgery from the patient perspective.

A 'core' outcome set for obesity surgery

As described above, heterogeneity of PROs in obesity surgery studies is an issue, which limits cross-study comparison and amalgamation of study results, which in turn limits the ability to make recommendations to clinicians and policymakers about the impact of obesity surgery on outcomes

For peer review only - http://bmjopen.bmj.com/site/about/guidelines.xhtml

important to patients. This issue is not unique to PROs, but also to clinical outcome measures with a Cochrane review of obesity surgery unable to undertake a meta-analysis due to a lack of consistency in outcomes reported[5].

Recent years have seen an increasing interest in the establishment of 'core outcome measures' to promote consistency in the reporting of clinical trials. Of particular interest is the development of the COMET (Core Outcome Measures in Effectiveness Trials) initiative through the MRC Hubs for Trials Methodology Research whose aim is to bring together researchers interested in the development and application of core outcome sets for specific conditions[9;10](1). Another notable initiative is OMERACT (Outcome Measures in Rheumatology Clinical Trials) in rheumatology[11]. Core outcome sets are not meant to be a restrictive list, but a minimum set of outcomes that should be reported on in every trial of a particular disease/condition. By standardising outcomes to be reported on in trials, results can be compared, contrasted and synthesised (e.g. meta-analysis) to inform health policy. No core outcome set for obesity surgery currently exists.

Recent research demonstrates that patients' perceptions of important outcomes may differ considerably from clinicians' perspectives, and therefore incorporating the patient perspective is crucial to contribute to and validate core outcome sets[12]. Creating a core outcome set for obesity surgery based on both health professional and patient views will help in the ordering of research and health service priorities to include the patient perspective, and ultimately improve long-term outcomes.

The patient perspective of follow-up care after obesity surgery

Follow-up care after obesity surgery varies greatly across centres. There is no consensus as to what optimum follow-up care is, although most clinicians recognise that good follow-up care is integral in achieving good outcomes from obesity surgery[13-15]. Understanding the outcomes of most importance to patients, and their experiences of follow-up care would provide invaluable information to design effective follow-up care programmes tailored to patients needs to maximise the benefits of surgery in the long-term. Qualitative research with obesity surgery patients in the UK context has focused on the experiences of patients, including difficulties they encounter after obesity surgery[16;17] but to our knowledge, no studies have specifically focused on patients' experiences of follow-up care and ways to improve follow-up care in the health services.

There is clearly more work needed to clarify optimum follow-up care from the patient perspective, and its influence on outcomes. As obesity and the number of obesity surgery operations increase, it is imperative that research in this area is of high quality, and takes into account the patient perspective.

Aims

The overall aims are to develop a core outcome set for obesity surgery including both health professional and patient perspectives and to investigate patients' experiences of living with the results of obesity surgery including ways to improve post-operative follow-up care.

Objectives

1. Semi-structured qualitative interviews with obesity surgery patients will be conducted to:
    a. Investigate outcomes of importance to obesity surgery patients that may not have been documented in the literature previously, and to add these to a comprehensive list of PROs of obesity surgery previously compiled from systematic literature reviews.
    b. Investigate patients' experiences of living with obesity surgery and ways to improve post-operative support.

For peer review only - http://bmjopen.bmj.com/site/about/guidelines.xhtml

2. The list created in objective 1a above will be added to a list of clinical outcomes which will be used within a Delphi survey of health professionals and patients which will ask them to prioritise the outcomes and produce a shorter list. The short list will be considered at separate meetings with health professionals and patients who will agree and finally ratify the content of the core outcome set.

Methods

Semi-structured qualitative interviews

To supplement the literature reviews previously undertaken by the lead researcher, patients' views of important outcomes of obesity surgery and experiences of existing follow up care will be investigated using semi-structured interviews within a qualitative paradigm.

Identification and selection of patients
Patients who are about to undergo and who have undergone a primary operation for obesity will be identified by health professionals from participating obesity surgery services at NHS healthcare Trusts using departmental databases and clinic lists. The main criteria for selection will be: The patient is undergoing obesity surgery within the next three months (pre-surgery group), or has undergone obesity surgery (post-surgery group). Including patients at both the pre- and post-operative stage will allow us to compare views and assess differences. Patients will initially be purposively sampled to obtain maximum variation for gender, age, ethnicity, starting BMI, type of operation, and time since operation, however further sampling will be guided by emerging findings from interviews.

Patient recruitment
Identified patients will be sent a letter from their local NHS obesity surgery team including an invitation letter and patient information sheet from the lead researcher, informing them of the research and inviting them to participate in the interviews. A reply slip and stamped addressed envelope will be included for patients to indicate whether or not they are interested in participating in the research to post back to the lead researcher. The number of patients recruited will depend on when theoretical saturation is reached (i.e. when the themes relevant to the research have been thoroughly investigated); however it is estimated that approximately 30 patients will be interviewed in total.

Data collection and analysis
Patients who agree to take part in the interviews will be able to choose the location of the interview, either in their own home, at one of the two participating hospitals, or the University of Bristol. Should any participants request to be interviewed at their GP surgery instead, permission will be sought by the chief investigator from the surgery's practice manager to conduct the interview at the surgery. Any travel costs will be reimbursed. Consent will be obtained face-to-face prior to the interview beginning. Should any participants request to be interviewed via telephone, participants will be mailed two copies of the consent form and asked to sign and return them to the chief investigator who will then sign them and return one copy to the participant prior to the interview. An interview topic guide will be applied flexibly to guide interviews. The following broad themes will be explored in interviews: 1) Motivations to undergo surgery; 2) Expected outcomes of surgery; 3) Actual outcomes of surgery (post-op group only); 4) Expectations of follow-up care; 5) Actual experiences of follow-up care (post-op group only). It is anticipated that interviews will last approximately 60 minutes, including 10 minutes dedicated to recording socio-demographic and relevant clinical data at the end of the interview. Interviews will be audio-recorded and transcribed in full. Separate consent will be sought to potentially re-contact them in the future for a follow-up interview.

For peer review only - http://bmjopen.bmj.com/site/about/guidelines.xhtml

Interviews will be analysed thematically using a grounded theory approach[18]. Data will be analysed using descriptive and explanatory coding to explore and inter-relate categories arising in the data. Data management will be facilitated using the program NVivo 9[19]. Descriptive accounts will be written up relating to each batch of interviews, and matrices will be drawn up to compare the occurrence of themes across interviews. Data analysis will run in parallel with data collection so that emerging themes can be followed up to enrich subsequent interviews. A small sample of the interview transcripts will be independently coded by both the chief investigator and her primary supervisor. The coding structures will then be discussed in a supervision meeting and revised as appropriate. The analysis will also be reviewed with the chief investigator's co-supervisor, and patient research partners.

Findings from the qualitative interviews will be combined with findings from a previous review of qualitative literature in this area, and written up for publication as soon as possible. Particular attention will be paid to ensuring that recommendations relating to the improvement of follow-up care are disseminated as soon as possible to clinicians and policymakers.

Important outcomes identified by patients in the semi-structured interviews will be added to the outcomes previously identified from systematic literature reviews undertaken by the lead researcher to create a comprehensive 'long' list of outcomes important to obesity surgery patients. The list will be reviewed by the lead researcher, her supervisors, and patient research partners to make sure it is comprehensive such that potentially important outcomes have not been omitted. This will form the basis of the next stage of the research (Delphi process).

Delphi process

This comprehensive list of outcomes will be refined into a 'core' outcome set using a Delphi process consisting of two questionnaire rounds where health professionals and patients individually rate each outcome for its value of being included in the core outcome set, followed by consensus meetings to discuss any potential areas of disagreement with regard to which outcomes to include and to ratify the final core outcome set.

Ethical issues arising

Informed consent

Written, informed consent will be obtained from all participants prior to the qualitative interviews. Confidentiality and anonymity will be assured at this stage and the purposes and possible uses of the research will be explained. It will be made clear that participants are free to drop out or delay participation in research at this stage. Separate consent will be sought to digitally-record interviews, and to potentially re-contact the participants in future for a follow-up interview.

Data protection

The principles of the Data Protection Act (1998) will be complied with and data obtained from interviewees will be anonymised using unique study codes. Documents to interpret the codes and personal data will be stored in separate encrypted files in separate locations on the University of Bristol server. All hard copy study documents will be stored in locked filing cabinets. Only data necessary to the purposes of the research will be obtained and stored.

For peer review only - http://bmjopen.bmj.com/site/about/guidelines.xhtml

Confidentiality

Confidentiality and anonymity outside of the interviews will be discussed and assured prior to beginning each interview. It will also be assured through the processes of data storage outlined above and the use of only fully anonymised quotations in all presentations of research findings.

Protection from distress

It is understood that living with morbid obesity and making the decision to undergo obesity surgery are personal and sensitive issues for patients. Therefore interviews will be undertaken sensitively and patients will only be encouraged to talk about aspects of their illness and care that they feel comfortable with. In the event that a participant becomes distressed during a research interview, the researcher will offer to take a short break from the interview and will remind participants that they do not have to discuss any topics they are uncomfortable with and are free to withdraw their consent to participate in the research at any time. If any patients express particular distress relating to their condition or treatment, a distress protocol will be followed (Appendix 1). The interviewer previously worked as a health professional in a NHS obesity surgery service and has experience of dealing with sensitive issues that may be brought up by this patient population. In addition, the interviewer will be supervised by a trained qualitative researcher who has experience in interviewing morbidly obese patients.

Integrity of research data

The MRC principles of Good Research Practice will be adopted with all primary data being retained so that there is an auditable trail from results back to data. Results will be fully and accurately reported at the end of the project.

Independence and impartiality

Every attempt will be made to maintain reflexivity at every stage of the research and to acknowledge the potential impact of the researcher upon the findings.

For peer review only

For peer review only - http://bmjopen.bmj.com/site/about/guidelines.xhtml

Appendix 1: Distress protocol - Interviews

All interviews will be prefaced with a statement about confidentiality and the duty of care. Participants will be told that interview is strictly confidential but should they disclose information to suggest that they are at significant risk of harm the researcher *may* have to discuss this with a clinical advisor.

In the event that a participant appears to be distressed during the interview (eg. becomes silent, cries) or discloses information to provoke concern about suicide risk, the following procedures will be followed:

- Participants will be offered the opportunity to pause for a break from the interview and will then be asked if they would like to resume.
- If necessary, the interview will be terminated and recording equipment stopped.
- At first, the interviewer will listen to the interviewee and offer support in situ. This will allow the researcher to assess whether further action is necessary.
- Should the interviewer remain concerned, they will reflect this to the interviewee and depending on the nature of the situation:
    1. Offer information about local help services
    2. Ask the interviewee if there is anyone they should contact, and if so attempt to make contact
    3. Offer to make initial contact with clinical services (primary or secondary) on behalf of the individual and with their consent
- In cases of particular concern the interviewer will
    1. If necessary, remain with the person until their distress has subsided or someone else is present
    2. Contact a local study clinician for advice/ assistance
    3. Provide a written report of the incident to AOS/JB (PhD supervisors), including information about the nature of the distress and the actions taken

Interviewees will be advised to contact their GP should they find subsequently that the interview provokes issues that they need to discuss.

For peer review only - http://bmjopen.bmj.com/site/about/guidelines.xhtml

Reference List

(1) The NHS Information Centre, Lifestyles Statistics. Statistics on obesity, physical activity and diet: England, 2012. The Health and Social Care Information Centre.

(2) World Health Organization. Fact sheet No. 311: Obesity and overweight. 2006.

(3) Must A, Spadano J, Coakley EH, Field AE, Colditz G, Dietz WH. The disease burden associated with overweight and obesity. *JAMA* 1999; 282(16):1523-1529.

(4) Buchwald H, Oien DM. Metabolic/bariatric surgery Worldwide 2008. *Obesity Surgery* 2009; 19(12):1605-1611.

(5) Picot J, Jones J, Colquitt JL, Gospodarevskaya E, Loveman E, Baxter L et al. The clinical effectiveness and cost-effectiveness of bariatric (weight loss) surgery for obesity: a systematic review and economic evaluation. *Health Technology Assessment* 2009; 13(41):1-+.

(6) U.S.Department of Health and Human Services FDA Center for Drug Evaluation and Research, U.S.Department of Health and Human Services FDA Center for Biologics Evaluation and Research, U.S.Department of Health and Human Services FDA Center for Devices and Radiological Health. Guidance for industry: patient-reported outcome measures: use in medical product development to support labeling claims: draft guidance. *Health & Quality of Life Outcomes* 2006; 4:79.

(7) Lasch KE, Marquis P, Vigneux M, Abetz L, Arnould B, Bayliss M et al. PRO development: rigorous qualitative research as the crucial foundation. *Quality of Life Research* 2010; 19(8):1087-1096.

(8) Coulman KD, Abdelrahman T, Owen-Smith A, Andrews RC, Welbourn CR, Blazeby JM. Measuring patient reported outcomes in bariatric surgery: standards of reporting and synthesis with clinical data. *Br J Surg* 2012; 99(Suppl. 2):1.

(9) University of Liverpool. COMET Initiative: http://www.comet-initiative.org/. 2012.

(10) Medical Research Council. MRC Hubs for Trial Methodology Research: http://www.methodologyhubs.mrc.ac.uk/. 2012.

(11) Tugwell P, Boers M, Brooks P, Simon L, Strand V, Idzerda L. OMERACT: An international initiative to improve outcome measurement in rheumatology. *Trials* 2007; 8.

(12) Hewlett SA. Patients and clinicians have different perspectives on outcomes in arthritis. *Journal of Rheumatology* 2003; 30(4):877-879.

(13) Myers VH, Adams CE, Barbera BL, Brantley PJ. Medical and Psychosocial Outcomes of Laparoscopic Roux-en-Y Gastric Bypass: Cross-sectional Findings at 4-Year Follow-up. *Obesity Surgery* 2012; 22(2):230-239.

(14) American Society for Metabolic & Bariatric Surgery. ASMBS Position Statement on Global Bariatric Healthcare. 2011.

For peer review only - http://bmjopen.bmj.com/site/about/guidelines.xhtml

(15) Heber D, Greenway FL, Kaplan LM, Livingston E, Salvador J, Still C. Endocrine and Nutritional Management of the Post-Bariatric Surgery Patient: An Endocrine Society Clinical Practice Guideline. *Journal of Clinical Endocrinology & Metabolism* 2010; 95(11):4823-4843.

(16) Ogden J, Clementi C, Aylwin S. The impact of obesity surgery and the paradox of control: A qualitative study. *Psychology & Health* 2006; 21(2):273-293.

(17) Ogden J, Clementi C, Aylwin S, Patel A. Exploring the impact of obesity surgery on patients' health status: a quantitative and qualitative study. *Obesity Surgery* 2005; 15(2):266-272.

(18) Glaser B, Strauss A. The discovery of grounded theory: Strategies for qualitative research. Chicago: Aldine; 1967.

(19) NVivo 9, QSR International; 2010.

For peer review only

For peer review only - http://bmjopen.bmj.com/site/about/guidelines.xhtml

Document S2: Topic guide

Post-operative patient interviews

NB This guide is necessarily provisional, as its application will depend on the experience of individual participants.

Introduction

- *Researcher explains research, asks if any queries on PIS, and takes consent.*

Motivations to undergo surgery

- To start off, I was just wondering if you could tell me a bit about what factors led to your decision to go for surgery?

Expected outcomes of surgery

- If you can think back to a few weeks before you had the surgery, can you remember how you were feeling about having surgery?
  Probe: Was there anything you were worried about? [Prompt on risks/side-effects of surgery]
  Probe: Was there anything you hoped would change for the better by having the surgery?
  Probe: Was there anything you were hoping to prevent by having surgery?

Actual outcomes of surgery

- How do you feel about having had surgery now?
  Probe: Which operation did you have?
  Probe: How long ago was it now?
  Probe: What's happened since? [Prompt on the things they said they were worried about/hoped would change/hoped they would prevent by having surgery]
  Probe: Was there anything that happened that you didn't expect? [Prompt on during surgery and after surgery]
  Probe: Are you glad you had the surgery?
  Probe: Do you think having the operation has changed your relationships with people who are important to you?
- To you, what are the most important results of surgery?
  If they say weight loss, Probe: What does weight loss mean for you?
  Probe: Are these things different to what you would have said before having surgery?
  Probe: What results of surgery might be most important to other people undergoing the surgery?
  If yes, Probe: How are they different?
- Are there any things that you didn't know beforehand that you wished you had?
- What do you think will happen in the future?

Expectations of follow-up care

- If you can think back to a few weeks before you had the surgery, can you remember if you had any expectations or ideas about the care you would receive after surgery? *[Prompt on short-term inpatient care and long-term care]*

Actual experiences of follow-up care

- Since you've had your surgery, have you been back to the hospital? *[Prompt on the surgical and weight management teams,]*
  Probe: What about your GP practice, have you seen anyone there since your surgery?
  Probe: Do you think there is a role for more follow-up care from primary care?
- What do you think will happen next?
- How do you feel about the NHS care you have received since your surgery?
  Probe: Tell me about the things you found most helpful
  Probe: Tell me about the things you found least helpful
  Probe: Is there anything you would have preferred to have been done differently?/Is there anything you think the NHS could have done to better support you since you've had your surgery?
- Could you say what you thought the most important things of good care after weight loss surgery were?
  Probe: Who would be the best person/people to do that?
  Probe: What would be the best setting for this? [Prompt on hospital/ another setting]
  Probe: Do you think these are the same things that others who've had surgery would say?
- What about support groups – have you been involved with any of these?
  If yes Probe: Do you tend to go to meetings or do you use online forums?
  Probe: How do you find these? [Prompt on positive and negative aspects]
  If no Probe: What's stopped you from getting involved?[ie. prompt on if unaware of them, none convenient, don't find helpful]
  If used to be involved but now no longer involved Probe: What's led to this?

Version 1.2 (18/3/2013)

For peer review only - http://bmjopen.bmj.com/site/about/guidelines.xhtml

**Clarifications, etc.**<br>❖ *Researcher asks participant to provide more information where points of interest were not previously probed.*
**Wind-down**<br>❖ *Researcher checks topic guide for omissions and prompts informant back towards areas of discussion where they seemed most comfortable.*
**Case report form**<br>❖ *Researcher records relevant socio-demographic and clinical details using the case report form.*
**Closing**<br>❖ *Researcher asks if patient would be interested in being contacted about a possible follow-up interview – to be recorded on consent form*<br>❖ *Researcher thanks interviewee for participating and asks if they would like a summary of the study results once complete*

For peer review only

For peer review only - http://bmjopen.bmj.com/site/about/guidelines.xhtml

Document S3: Case Report Form - Patient views about weight loss surgery

Participant identification number:<br>Date of birth: __/__/____<br>Initials:<br>Today’s date: __/__/____	*If patient has consented to GP being informed about participation in study*:<br>GP name:<br>GP surgery and address:

1.	What is the gender of the participant? 1=Male, 2=Female	☐
2.	What is the patient’s ethnicity? 1=White British, 2=White other, 3=Black, 4=Asian, 5=Mixed, 6=Chinese, 7=Other	☐
3.	Who does the participant live with? 1=alone, 2=with family, 3=with other adults	☐
4.	What is the participant’s marital status? 1=single, 2=married, 3=separated, 4=divorced, 5=widowed	☐
5.	What is the highest level of education completed? 1=less than compulsory school education, 2=compulsory school education, 3=post-compulsory school education below university level, eg. advanced technical school/advanced vocational, 4=university level	☐
6.	What is the employment status of the participant? 1=employed full-time (could be on sick leave), 2=employed part-time, 3=homemaker, 4=student, 5=unemployed, 6=retired, 7=self-employed, 8=other (specify)...	☐
7.	What is the current or last occupation of the participant?...	
8a.	What is the patient’s surgical status? 1=Awaiting surgery, 2=Undergone surgery (go to 8e.)	☐
b.	If the participant is awaiting surgery, what operation are they hoping to undergo? 1=LAGB, 2=RYGB, 3=SG, 4=Other (specify)..	☐
c.	If the participant is awaiting surgery, do they have a date? 1=Yes, 2=No	☐
d.	If yes, what is the date?	__/__/____
e.	If the participant has undergone surgery, what operation have they undergone? 1=LAGB, 2=RYGB, 3=SG, 4=Other (specify)..	☐
f.	If the participant has undergone surgery, what date did it occur?	__/__/____
9a.	What was/is the participant’s weight prior to surgery (self-reported)?	☐ Stones ☐ lbs ☐ Kg
b.	If the participant has undergone surgery, what is their weight now (self-reported)?	☐ Stones ☐ lbs ☐ Kg
10.	What is the participant’s height (self-reported)?	☐ feet ☐ inches ☐ cm

For peer review only - http://bmjopen.bmj.com/site/about/guidelines.xhtml

For peer review only

11.	Does the participant have any co-morbidities? List as many as needed. 1=diabetes, 2=hypertension, 3=hyperlipidaemia, 4=cardiac disease (excluding 2 and 3), 5=sleep apnoea, 6=asthma, 7=joint problems (eg. arthritis), 8=urinary incontinence, 9=infertility, 10=other, 11=None. If other, please specify...	☐ ☐ ☐ ☐

For peer review only

For peer review only - http://bmjopen.bmj.com/site/about/guidelines.xhtml

For peer review only

Document S4: Final coding structure used in NVivo

Name of code	Code related to
General NHS comments not related to surgery	Aspects of care
Guidelines from health professionals	Aspects of care
Follow-up care	Aspects of care
Factors leading to decision to have surgery	Life pre-surgery
Past struggles with weight	Life pre-surgery
Pre-op feelings about surgery	Life pre-surgery
Expectations of surgery	Life pre-surgery
Pre-surgery preparation	Life pre-surgery
Liver shrinking pre-op diet	Life pre-surgery
Other people's perceptions of obesity	Life pre-surgery
NHS funding of surgery	Life pre-surgery
Criteria to fulfill to obtain surgery	Life pre-surgery
Background	Life pre-surgery
Choice of operation	Life pre-surgery
Social impact of obesity	Life pre-surgery
Treatment from health professionals around obesity	Life pre-surgery
Other people's experiences of surgery	Life post-surgery
Future expectations and hopes	Life post-surgery
Peri-operative experience	Life post-surgery
Factors that help maintain motivation post-surgery	Life post-surgery
Support groups	Life post-surgery
Outcome - Activity, mobility	Life post-surgery
Outcome - Body image	Life post-surgery
Outcome - Comorbidity	Life post-surgery
Outcome - Eating	Life post-surgery
6 week post-op diet	Life post-surgery
Outcome - GI symptoms	Life post-surgery
Bowel movements	Life post-surgery
Wind or gas	Life post-surgery
Dumping or sickness	Life post-surgery
Outcome - Hunger	Life post-surgery
Outcome - Psychological	Life post-surgery
Outcome - Social impact of surgery	Life post-surgery
Family reactions to surgery	Life post-surgery
Outcome - Weight	Life post-surgery
In-hospital side-effects of surgery	Life post-surgery
Post-discharge side-effects of surgery	Life post-surgery
Most important outcomes of surgery	Life post-surgery
Outcome - Normality	Life post-surgery
Outcome - Clothing	Life post-surgery
Outcome - increased work options	Life post-surgery

For peer review only

COREQ checklist

No	Item	Guide questions/description	Location in text
**Domain 1: Research team and reflexivity**			
Personal characteristics			
1.	Interviewer/facilitator	Which author/s conducted the interview or focus group?	Page 7, lines 8-9
2.	Credentials	What were the researcher's credentials? E.g. PhD, MD	Page 7, line 9
3.	Occupation	What was their occupation at the time of the study?	Page 7, line 9
4.	Gender	Was the researcher male or female?	Page 7, line 9
5.	Experience and training	What experience or training did the researcher have?	Page 7, lines 10-11
Relationship with participants			
6.	Relationship established	Was a relationship established prior to study commencement?	Page 7, lines 11-13
7.	Participant knowledge of the interviewer	What did the participants know about the researcher? e.g. *personal goals, reasons for doing the research*	Page 7, lines 13-15
8.	Interviewer characteristics	What characteristics were reported about the interviewer/facilitator? e.g. *Bias, assumptions, reasons and interests in the research topic*	Page 7, lines 8-15; page 8 lines 12-13
**Domain 2: Study design**			
Theoretical framework			
9.	Methodological orientation and Theory	What methodological orientation was stated to underpin the study? e.g. *grounded theory, discourse analysis, ethnography, phenomenology, content analysis*	Page 7, lines 18-19

For peer review only

Participant selection			
10.	Sampling	How were participants selected? e.g. purposive, convenience, consecutive, snowball	Page 6, lines 14-16
11.	Method of approach	How were participants approached? e.g. face-to-face, telephone, mail, email	Page 6, lines 12-14
12.	Sample size	How many participants were in the study?	Page 8, line 20
13.	Non-participation	How many people refused to participate or dropped out? Reasons?	Page 8, line 20
Setting			
14.	Setting of data collection	Where was the data collected? e.g. home, clinic, workplace	Page 7, line 1-3
15.	Presence of non-participants	Was anyone else present besides the participants and researchers?	Page 8, line 21-22
16.	Description of sample	What are the important characteristics of the sample? e.g. demographic data, date	Page 8 lines 20-24; Table 1
Data collection			
17.	Interview guide	Were questions, prompts, guides provided by the authors? Was it pilot tested?	Page 7, lines 4-7; Page 8, lines 15-17; Document S2
18.	Repeat interviews	Were repeat interviews carried out? If yes, how many?	Page 7, lines 11-12
19.	Audio/visual recording	Did the research use audio or visual recording to collect the data?	Page 7, lines 8-9
20.	Field notes	Were field notes made during and/or after the interview or focus group?	Page 7, lines 15-16
21.	Duration	What was the duration of the interviews or focus group?	Page 7, line 3

For peer review only

22.	Data saturation	Was data saturation discussed?	Page 6, lines 17-18
23.	Transcripts returned	Were transcripts returned to participants for comment and/or correction?	Page 7 line 17.
**Domain 3: Analysis and findings**			
Data analysis			
24.	Number of data coders	How many data coders coded the data?	Page 7, lines 21-23
25.	Description of the coding tree	Did authors provide a description of the coding tree?	Page 7, line 23; Document S4
26.	Derivation of themes	Were themes identified in advance or derived from the data?	Page 7, lines 18-21
27.	Software	What software, if applicable, was used to manage the data?	Page 7, line 7; page 8, line1
28.	Participant checking	Did participants provide feedback on the finding?	Page 8, lines 15-18
Reporting			
29.	Quotations presented	Were participant quotations presented to illustrate the themes/findings? Was each quotation identified? *eg. participant number*	Pages 9-14
30.	Data and findings consistent	Was there consistency between the data presented and the findings?	Pages 9-14
31.	Clarity of major themes	Were major themes clearly presented in the findings?	Pages 9-14; Figures 1 and 2
32.	Clarity of minor themes	Is there description of diverse cases or discussion of minor themes?	Pages 9-14; Figures 1 and 2

For peer review only
